# SuperFeat: Quantitative Feature Learning from Single-cell RNA-seq Data Facilitates Drug Repurposing

**DOI:** 10.1093/gpbjnl/qzae036

**Published:** 2024-05-23

**Authors:** Jianmei Zhong, Junyao Yang, Yinghui Song, Zhihua Zhang, Chunming Wang, Renyang Tong, Chenglong Li, Nanhui Yu, Lianhong Zou, Sulai Liu, Jun Pu, Wei Lin

**Affiliations:** State Key Laboratory for Oncogenes and Related Genes, Department of Cardiology, Renji Hospital, School of Medicine, Shanghai Jiao Tong University, Shanghai Cancer Institute, Shanghai 200127, China; Department of Laboratory Medicine, Xin Hua Hospital, School of Medicine, Shanghai Jiao Tong University, Shanghai 200092, China; Central Laboratory of Hunan Provincial People’s Hospital/The First Affiliated Hospital of Hunan Normal University, Changsha 410005, China; Central Laboratory of Hunan Provincial People’s Hospital/The First Affiliated Hospital of Hunan Normal University, Changsha 410005, China; State Key Laboratory for Oncogenes and Related Genes, Department of Cardiology, Renji Hospital, School of Medicine, Shanghai Jiao Tong University, Shanghai Cancer Institute, Shanghai 200127, China; State Key Laboratory for Oncogenes and Related Genes, Department of Cardiology, Renji Hospital, School of Medicine, Shanghai Jiao Tong University, Shanghai Cancer Institute, Shanghai 200127, China; Central Laboratory of Hunan Provincial People’s Hospital/The First Affiliated Hospital of Hunan Normal University, Changsha 410005, China; Central Laboratory of Hunan Provincial People’s Hospital/The First Affiliated Hospital of Hunan Normal University, Changsha 410005, China; Central Laboratory of Hunan Provincial People’s Hospital/The First Affiliated Hospital of Hunan Normal University, Changsha 410005, China; Central Laboratory of Hunan Provincial People’s Hospital/The First Affiliated Hospital of Hunan Normal University, Changsha 410005, China; State Key Laboratory for Oncogenes and Related Genes, Department of Cardiology, Renji Hospital, School of Medicine, Shanghai Jiao Tong University, Shanghai Cancer Institute, Shanghai 200127, China; State Key Laboratory for Oncogenes and Related Genes, Department of Cardiology, Renji Hospital, School of Medicine, Shanghai Jiao Tong University, Shanghai Cancer Institute, Shanghai 200127, China

**Keywords:** Single-cell transcriptomics, Cell state transition, Cell scoring, Drug search, Feature learning

## Abstract

In this study, we devised a computational framework called Supervised Feature Learning and Scoring (SuperFeat) which enables the training of a machine learning model and evaluates the canonical cellular statuses/features in pathological tissues that underlie the progression of disease. This framework also enables the identification of potential drugs that target the presumed detrimental cellular features. This framework was constructed on the basis of an artificial neural network with the gene expression profiles serving as input nodes. The training data comprised single-cell RNA sequencing datasets that encompassed the specific cell lineage during the developmental progression of cell features. A few models of the canonical cancer-involved cellular statuses/features were tested by such framework. Finally, we illustrated the drug repurposing pipeline, utilizing the training parameters derived from the adverse cellular statuses/features, which yielded successful validation results both *in vitro* and *in vivo*. SuperFeat is accessible at https://github.com/weilin-genomics/rSuperFeat.

## Introduction

Since the emergence of various high-throughput omics technologies such as microarray [1] and next-generation sequencing (NGS) [2], researchers have been generating vast amounts of molecular profiling datasets of the biological and clinical samples at an unprecedented rate. The single-cell sequencing technique has even added another degree of magnitude and a new dimension of information [3–6]. Many models and algorithms have been developed to evaluate the biological signal and characterize the sample. Nonetheless, most of the existing methods are hardly generalizable but specific to particular problems [7,8]. A universal framework for the rapid yet generic assessment of the biological/clinical samples using molecular profiling data could be very useful and efficient, especially for the omics assays at high granularity.

Human learning involves the processes of classification and quantification. The artificial neural network (ANN) has been used as a flexible framework to simulate and streamline such processes using a machine/computer. It provides a relatively simple but generalizable classification and quantification model to automatically structure the human knowledge acquired from a large body of data. The ANN nodes of the neural network reflect the qualitative or the quantitative state of a biological concept in the human mind. Most importantly, subsequent human decisions, such as clinical decisions, could be made based on the evaluation of the cellular state. Therefore, the ANN structure could be used to realize the automatic learning and evaluation of biological statuses/features and thus facilitate making decisions more efficiently based on the input of a high volume of data from the high-throughput omics techniques. This mechanism is becoming increasingly powerful and can accomplish many unprecedented tasks, even in the biomedical field.

Previously, we developed the ANN-based cell type classifier framework, SuperCT [9]. It uses the single-cell RNA sequencing (scRNA-seq) digital expression profiles as input to characterize the canonical cell lineages. Similar cell type classification strategies have been published since then [10,11]. This work represents an early attempt to apply ANNs to high-throughput single-cell gene expression data, offering a general interface for the now prevalent deep learning models. In a fully connected neural network for a cell type classifier, the node weight reflects the empirical contribution of a gene to a certain cell type, which also makes this classifier interpretable.

In the meantime, a certain cell type could undergo a spectrum of variable cellular states reflected by a few function-related and signaling-related gene expression patterns. For instance, T cells turn into an exhaustion state in most solid tumors [12]; macrophages undergo polarization in certain biological contexts [13,14]. Nonetheless, assessing the variability of such cellular state by a couple of markers using the current single-cell transcriptomics data based on barcoded-bead (BCB) oftentimes is not robust. This is due to the stochastic nature of the RNA transcription within a single cell [15] and the limitation of detection for a specific molecule by the current BCB capture [4]. Unless we perform a more comprehensive assessment using a group of coordinately expressed genes, the state of a single cell might not be accurately evaluated. The good news is that a certain cellular status/feature is always associated with a group of up-regulated and down-regulated genes, which thus can be reflected by a scoring strategy based on the presence/absence of a group of transcriptions.

The strategy of activating or reversing a certain cellular state of the disease-involved cell type could be employed for possible therapeutics. For example, immune checkpoint inhibition has been used in cancer therapy. It serves the purpose of reversing the T cell exhaustion [16]. Targeting helper T cellular state conversion could be used in mitigating autoimmunity [17]. Ideally, if small molecules could be used to reverse the adverse cellular state or promote the beneficial cell state. Such an idea was implemented in the Connectivity Map (CMap) bulk (but not single-cell) transcriptomics datasets. Cell lines have been used to generate the CMap perturbagen transcriptomics data repository, which was used to search for repurposing drugs [18,19]. Nonetheless, prior to the application of the scRNA-seq technique to clinical samples, the CMap search was oftentimes compromised by the convoluted signals of a mixture of cell types of different roles. The high-resolution transcriptomics data like scRNA-seq allow to characterize the target cell population and thus make the CMap search more specific. signatureSearch provided a flexible tool to use ranked gene set to search a perturbagen database that could be used to achieve such goal [20].

In addition to the scRNA-seq, a similar BCB-based RNA quantification strategy is applied in the 10X Genomics Visium spatial transcriptomics (ST) platform. Obviously, the cellular status/feature evaluation strategy could be applicable to this type of data and provide a very user-friendly spatial visualization of the histological slides. The landscape of the cellular statuses/features will be smoothed even with the stochastic transcriptions of the functional genes on the spots.

In this study, to efficiently perform the learning and quantitative assessment of a variety of biological features documented in the literature, we built an ANN-based framework, Supervised Feature Learning and Scoring (SuperFeat). This framework can facilitate the thorough evaluation of the critical features of the tissue samples or even the precise diagnoses of the patients with adequate details. Considering that gene set-based methods such as Seurat modular score [21], AUCell [22], singscore [23], and gene set variation analysis (GSVA) [24] can somewhat do a similar job, we performed an extensive performance comparison to understand the strength of SuperFeat.

## Method

### Organization and preprocessing of the training and validation datasets

The datasets for feature training are summarized in Table S1. The cell populations with the designated cellular statuses/features came from the unsupervised clustering, and were enriched with the canonical markers for the specific status/feature. The annotations were conducted by the authors of the original papers. For the training dataset, we only included the cell types in which the statuses/features were supposed to be exhibited in the model training. A total of 19,202 genes (Table S2) were taken into the input, which were derived from the total genes of the MSigDB v7.4.1 gene sets of subcategories H and C5 [25]. The missing expression values of these 19,202 genes in the training or validation dataset were filled up with zero. The gene expression values of the digital expression matrix were transformed to a binary matrix.

### Implementation of the SuperFeat ANN

The ANN structures and the learning strategy were implemented using Python library keras v2.9.0 and sklearn v1.0.2. We designed a fully connected ANN model for cell type classification. Similar to what has been done for SuperCT, the inputs were transformed into the binary values of 19,202 genes. As seen in most of the flow cytometry analyses, the presence/absence of the signature gene partially contributes to the status/feature of the cell. Also, the binary signal input is compatible across most of the unique molecular index (UMI)-based scRNA-seq platforms with the robust performance of the cell type classification. The code could be found at https://github.com/weilin-genomics/rSuperFeat/blob/main/train_new_model/SuperFeat_trainingCode.py.

The input layer was connected to a hidden dense layer with only one hidden layer neuron using rectified linear unit (ReLU) activation functions. The single node in the hidden layer represented that a neuron received the signal from 19,202 inputs by different weights, only if the combined stimulation by these input signals was above a certain level, it determined the probability of the cell either in state0 cell or state1, by outputting two nodes. The input layer had the L1 regularization with a coefficient of 0.01. To avoid the under-representation of the small-sample-size cell types in the calculation of the accuracy function, we included the class weight based on the sample size of each type in the model training. The loss function was defined as categorical cross-entropy.

### Evaluation of the designated cellular features using SuperFeat score

The cellular status/feature was evaluated using the following equation:

(1) $$\mathrm{SuperFeat}\;\mathrm{score}=\sum_{n=1}^{19202}w_{i}{\;\cdot \;g}_{i}+b\;$$

where *w_i_* denotes the weight value of gene *i* from the model trained from the training dataset to the first layer, *g_i_* denotes whether gene *i* is detected in this cell or not, and *b* denotes the bias value for the single node of the first hidden layer.

### Drug search using weight-ranked genes

In this study, we utilized the qSig function from the signatureSearch package v1.8.2 to conduct connectivity ranking, drawing on the perturbational cell gene expression signatures within the CMap and LINCS L1000 databases [26]. The top 250 genes, both positively and negatively weighted, were identified using the printTopWeights function from the rSuperFeat package and subsequently input into “signatureSearch” to identify potential drugs. Our methodology incorporated reference databases from both CMap and LINCS, employing their respective gene expression signature search (GESS) methods.

Drug search was predicated on cumulative positive or negative connectivity scores, indicating their potential to either induce or counteract shifts in cellular states or features. For LINCS, we prioritized drug–cell type pairs using the weighted connectivity score (WTCS); while for CMap, ranking was based on the raw score metric. If the cellular transition is beneficial, we should prioritize the drug with larger positive scores. Conversely, if the cellular transition is detrimental, we should prioritize the drug with larger negative scores. When using “signatureSearch” for drug searching, different scores were employed due to the differences of two perturbagen databases, and the statistical assumptions satisfied by the analysis signals varied.

### Unsupervised clustering, dimensional reduction, and data visualization in scRNA-seq analyses

For the scRNA-seq dataset downloaded from Gene Expression Omnibus (GEO) database, the unsupervised clustering, dimensional reduction, and data visualization in this study were realized by a widely used scRNA-seq analytical suite, Seurat v4.1.0. The Seurat objects were generated for each dataset with their digital expression matrices as input. The principal component analysis (PCA) was performed by Seurat RunPCA function. The *t*-distributed stochastic neighbor embedding (*t*-SNE) coordinates were calculated using Seurat RunTSNE function. The uniform manifold approximation and projection (UMAP) coordinates were calculated using Seurat RunUMAP function. The putative clusters were defined by Seurat FindClusters function using the top 10 principal components and other default parameters. If the cell annotation was provided by authors in the database, we will directly use their annotation. If not, the unsupervised clusters were re-annotated according to the enriched literature markers. The receiver operating characteristic (ROC) analysis was implemented with pROC package v1.18.0 [27].

### Cell culture

The GC cell line MKN-45 was purchased from the Cell Bank of Type Culture Collection of the Chinese Academy of Sciences and cultured in Dulbecco’s Modified Eagle Medium (DMEM; Catalog No. 21063029, Gibco, Carlsbad, CA) with 10% phosphate-buffered saline (PBS) and antibiotics (100 IU/ml penicillin and 100 µg/ml streptomycin). The cell culture was placed in humidified air at 37°C with 5% CO_2_/95% air (v/v).

### *In vivo* subcutaneous tumor generation and drug treatment

For* in vivo* studies, 4–6-week-old male BALB/c nude mice (Shanghai Laboratory Animal Center, Shanghai, China) were housed in a controlled environment with a 12-h light/12-h dark cycle, with free access to water and food at temperature of 21°C–23°C and humidity of 40%–60%.

Low-passage MKN-45 cells were resuspended in a 1:1 mixture of PBS and Matrigel (Catalog No. 356231, Corning, NY) at 1 × 10^6^ cells/ml. Then, 100 µl of cell stock was injected subcutaneously on the shaved right flank of BALB/c nude mice. After 10 days of growing, the tumor volume increased to an average size of 60 mm^3^. WH-4023 (Catalog No. S7565, Selleck, Shanghai, China) was dissolved in dimethyl sulfoxide (DMSO; Catalog No. D2650, Sigma-Aldrich, Saint Louis, MO), and then injected into the tumors every other day for 6 days at the concentration of 0.5 mg/kg body weight. The same volume of DMSO was included as control. The mice were examined at regular time points until they were sacrificed. The tumor size was measured using a digital caliper, and the tumor volume was calculated with the following formula: volume = 0.5 × width^2^ × length.

### ST dataset

We conducted an in-depth ST study on samples from two cases of intrahepatic cholangiocarcinoma (iCCA) using the Visium technology from 10X Genomics. These slides were simultaneously subjected to hematoxylin and eosin (H&E) staining, and different histopathological regions were independently annotated by two distinct pathology experts for various stained areas.

#### Tissue processing

Tumors from two iCCA cases were washed with PBS, and any liquid was soaked up with gauze. Tissues were cut into pieces of 6.5 mm³, snap-frozen in isopentane cooled with liquid nitrogen, and stored at −80°C.

#### Slide preparation

Slides for ST had four 6.5 × 6.5 mm^2^ areas with 5000 barcoded primer spots. Tissue sections were cut at 10-μm thickness and placed on specific slides from 10X Genomics.

#### Fixation, staining, and imaging

Sectioned slides were fixed, stained with H&E, and mounted with glycerol. Bright-field images were taken at 20× magnification and processed with VSlide software [28].

#### Reverse transcription and sequencing

The protocol followed was as described using the Visium platform of 10X Genomics. Sequencing was performed on an Illumina NovaSeq 6000, and initial data analysis was conducted with Space Ranger software [29], mapping to the GRCh38_release 95 human genome.

### Immunofluorescence staining

The tumor tissues were harvested and fixed in 4% paraformaldehyde, embedded in paraffin, followed by cryosection with a thickness of 5–10 μm. The cryosections were fixed with 4% paraformaldehyde, permeabilized with 0.1% Triton X-100 in PBS for 15 min, and blocked with 10% fetal bovine serum (FBS)/PBS for 1 h. Then, the cryosections were stained with the primary antibodies: anti-CD34 (Catalog No. ab81289, Abcam, Waltham, MA), anti-PI16 (Catalog No. PA5-111740, Invitrogen, Carlsbad, CA), anti-COL1A1 (Catalog No. PA5-29569, Invitrogen), anti-α-SMA (Catalog No. ab124964, Abcam). The primary antibodies were diluted in 10% FBS/PBS by the dilution factor recommended by the suppliers, applied to the samples, and incubated at 37°C for 1.5 h or at 4°C overnight. The secondary antibodies were diluted at 1:1000 in 10% FBS/PBS, applied to the samples, and incubated at 37°C for 45 min. The cell nucleus was counterstained with 4’,6-diamidino-2-phenylindole (DAPI) at room temperature for 5 min, and coverslips were mounted on slides with fluorescent mounting media.

## Results

### An efficient framework of feature learning and scoring using single-cell transcriptomics data

**Figure 1:**
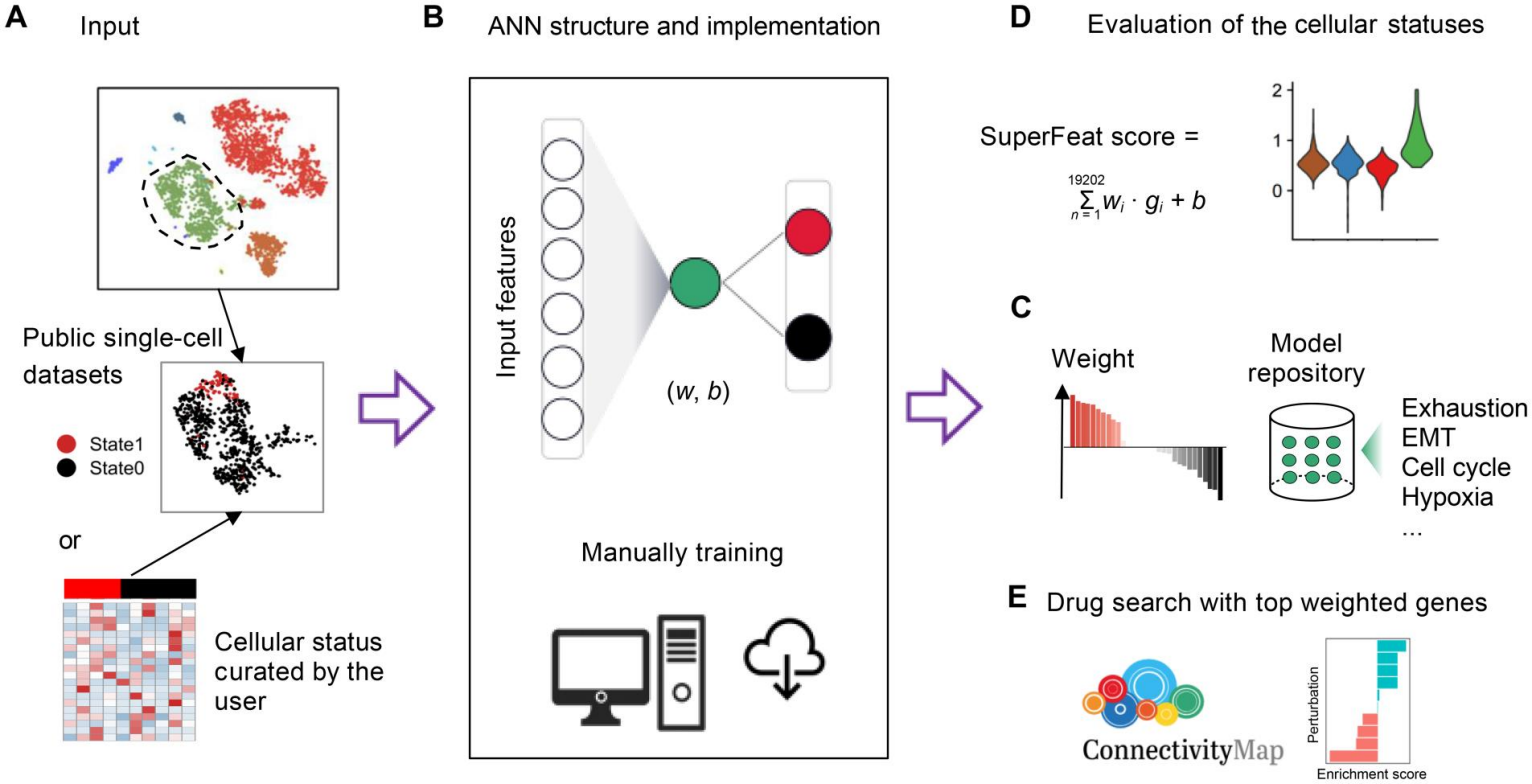
Workflow of SuperFeat framework **A**. A diagram showing how the cell lineage with a variable cellular status/feature is defined in the training dataset. **B**. ANN structure and implementation. **C**. The parameters of the models trained from dataset and the repository of models for the canonical cellular statuses/features. **D**. Evaluation of the cellular statuses/features using SuperFeat model and visualization. **E**. Drug search using the model parameters. scRNA-seq, single-cell RNA sequencing; EMT, epithelial-mesenchymal transition; ANN, artificial neural network.

We constructed a fully connected ANN, SuperFeat, to learn the quantitative features with one single node in the hidden layer and two output nodes. The value of the middle-layer node will be used for the quantitative evaluation of the cellular status/feature. **Figure 1** illustrates the workflow of the entire framework. Figure 1A shows a single-cell dataset containing multiple cell types. If one of the cell types (circled by the dashed line) was annotated with a variable cellular status/feature (according to some signature genes) and can be manually divided into two annotated subpopulations, this variable cell population could be used to train the SuperFeat model for evaluating this cellular status/feature. If there are more datasets with the similar cellular state annotation, we should include the cell populations from more datasets to enhance the representativity and the model generalizability. To enable users to customize their own model, we also provided the Python code (Figure 1B) for those who likes to train their own cellular status/feature model and to assess similar transcriptomics data. Figure 1B also shows the structure of the ANN, and the green node basically provides a score to evaluate how much the transcriptional profile should be categorized to state1 (in red) or state0 (in black).

Figure 1C shows the weights of the genes contributing to the score in this ANN model. Figure 1D shows how the model could be used to calculate the score of the cellular status/feature of the cells from another dataset of scRNA-seq or spatial RNA sequencing (RNA-seq) in the applicable biological context. This part of application was implemented in an R package rSuperFeat (https://github.com/weilin-genomics/rSuperFeat). Later, as shown in Figure 1E, the model parameters can be further utilized to search for the CMap [18] perturbagen that potentially enhances or alleviates the stress associated with cellular state change.

### Training and evaluation of four tumor hallmark cellular statuses/features

**Figure 2:**
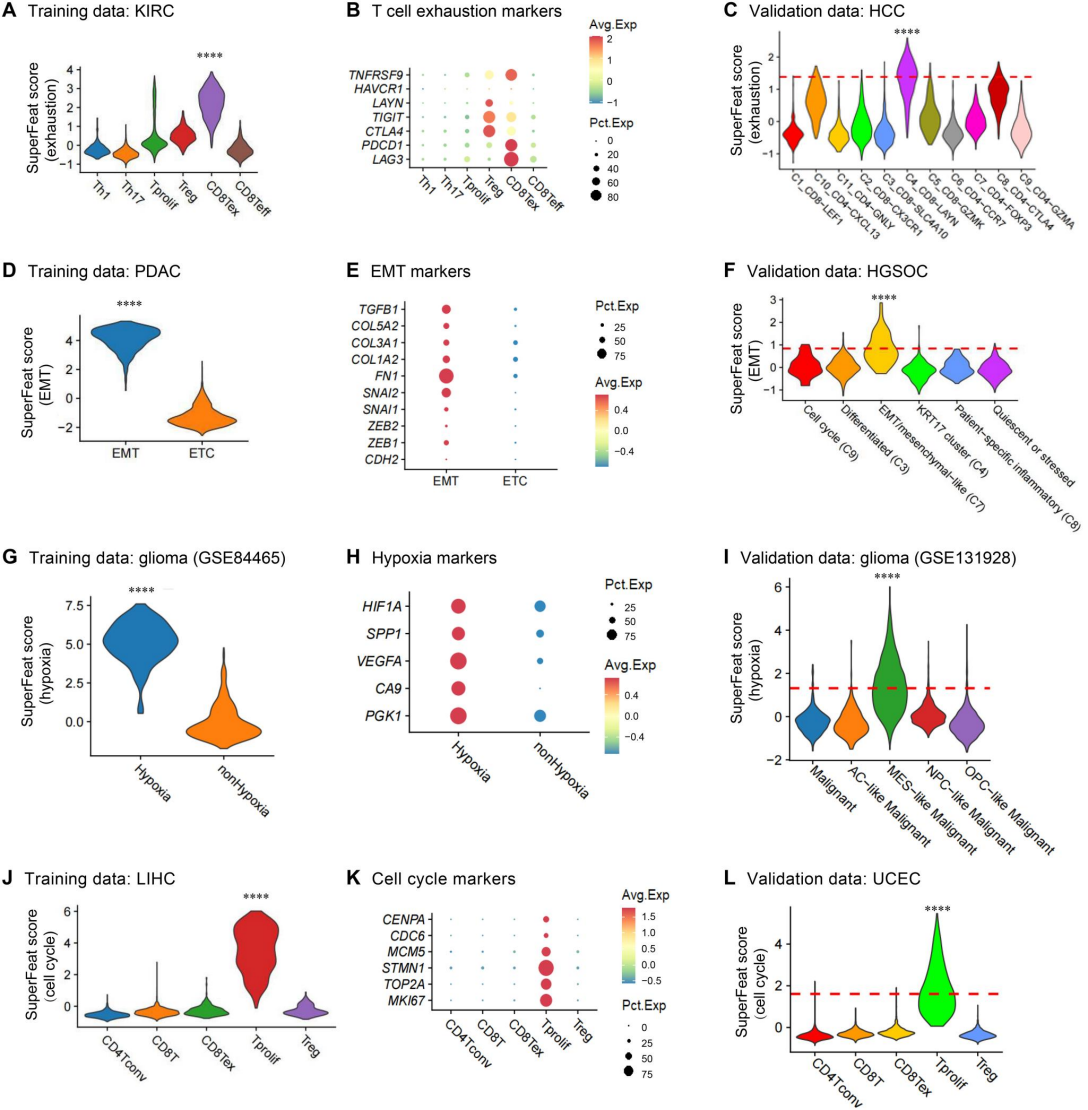
The tumor-related cellular statuses/features defined by SuperFeat **A**. T cell exhaustion SuperFeat scores on training data. **B**. Canonical T cell exhaustion markers. **C**. SuperFeat scores validated by a HCC dataset. **D**. EMT SuperFeat scores on training data. **E**. Canonical EMT markers. **F**. SuperFeat scores validated by a HGSOC dataset. **G**. Hypoxia SuperFeat scores on training data. **H**. Canonical hypoxia markers. **I**. SuperFeat scores validated by a glioma dataset. **J**. Cell cycle SuperFeat scores on training data. **K**. Canonical cell cycle markers. **L**. SuperFeat scores validated by a UCEC dataset. All *P* values were determined by Wilcoxon test (****, *P* < 0.0001). KIRC, kidney clear cell carcinoma; HCC, hepatocellular carcinoma; PDAC, pancreatic ductal adenocarcinoma; HGSOC, high-grade serous ovarian cancer; LIHC, liver hepatocellular carcinoma; UCEC, uterine corpus endometrial carcinoma.

Using our most recent version of SuperFeat training framework and the training dataset from a study of kidney renal clear cell carcinoma (KIRC) [30], we first performed the training of the T cell exhaustion model parameters and applied the scoring model to assess the T cells. The exhaustion scores allowed us to discern the exhausted and active T/natural killer cell populations that were correlated to the immunotherapy. The distributions of the exhaustion scores of the annotated populations of T cells in training dataset are shown in **Figure 2**A. The layout of these cells is shown in Figure S1. The canonical marker signals of exhaustion annotated by Neal et al. [30] are shown in Figure 2B, confirming the overall concordance. Then we used the datasets including the infiltrating T cells from hepatocellular carcinoma (HCC) samples published by Zheng et al. [31] to test the model performance. The C4_CD8-*LAYN* population stood out (Figure 2C), which was concordant to the cluster interpretation by Zheng and colleagues [31]. The layout of the cell populations in the validation datasets is shown in Figure S2.

Epithelial-mesenchymal transition (EMT) is another example of the variable cellular state that oftentimes occurs in the epithelial cells under wound healing, organ fibrosis, and especially initiation of the metastasis of cancer progression. Here, we used a set of pancreatic ductal adenocarcinoma (PDAC) single-cell data with EMT annotation in the tumors [32] to train a EMT model. We used the EMT population from high-grade serous ovarian tumor [33] to test the performance of this model. The scores of the training cells are shown in Figure 2D. The C7 cluster, which was annotated as “EMT/mesenchymal-like” population, exhibited higher EMT signals. Both show a concordance with the published annotation and the canonical EMT markers (Figure 2E). The scores of the testing cells are shown in Figure 2F.

Hypoxia refers to a state in when the cells are deprived of adequate oxygen. Tumors oftentimes develop such a hypoxic feature. We used Darmanis et.al. dataset [34] to train the hypoxia model and used Neftel et.al. dataset [35] to evaluate the feature. The results are shown in Figure 2G–I. The annotated hypoxic cell populations (the MES-like malignant cluster) in the validation dataset gave high SuperFeat scores and exhibited canonical hypoxic gene expression such as *HIF1A* and *VEGFA*.

The proliferation rate is essential to the development of tissue. For example, the immunohistochemistry (IHC) signature Ki-67 (with gene symbol *MKI67*) has been commonly used to evaluate cell proliferation by pathologists. Here, we also trained a model of proliferation [36] and tested this model performance [37]. Figure 2J shows the proliferation scores in the training dataset, and Figure 2K shows the transcriptional signals of the canonical cell cycle genes. The proliferation scores of the testing cells are shown in Figure 2L.

### SuperFeat model parameters uncover involved known and novel features

The neural network used to be considered a black-box learning machine. As our framework has a very simple network structure with a limited number of nodes in the middle layer and the input genes are trained independently, the contribution of the input nodes (genes) can be easily evaluated by the weight values. Therefore, the top positively/negatively weighted genes could be used to perform an enrichment analysis to understand how they underlie the cellular state change.

**Figure 3:**
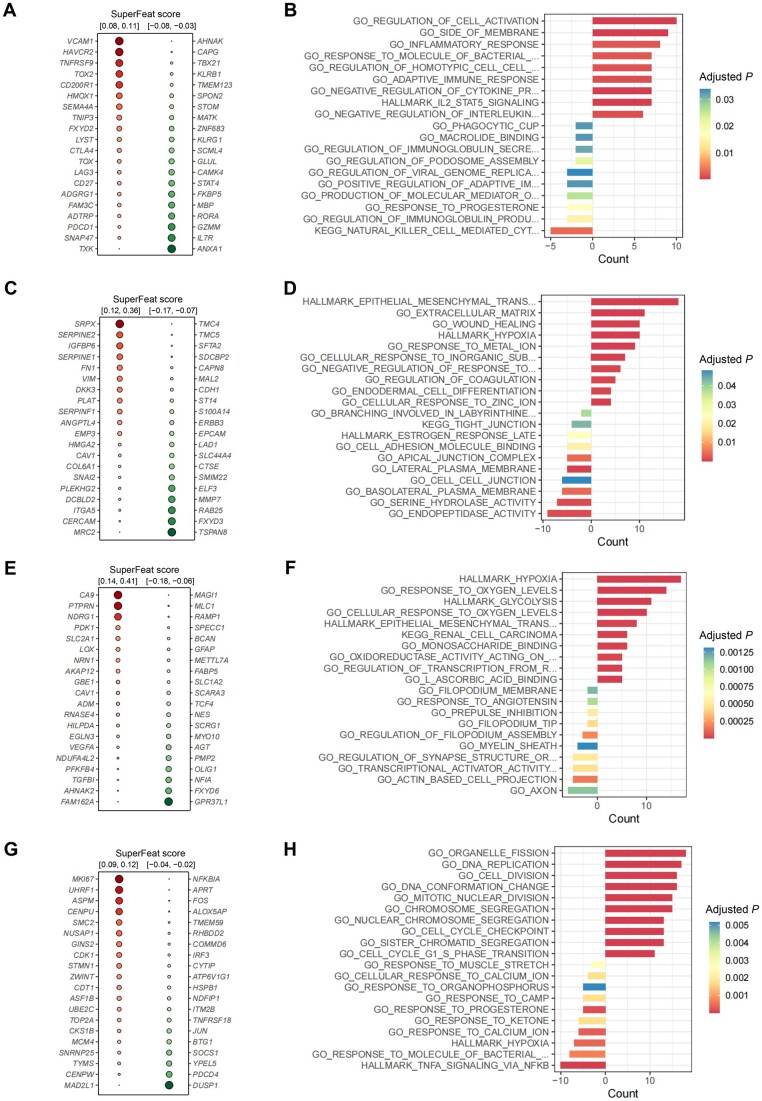
Interpretation of SuperFeat model parameters **A**. Top 20 positively/negatively weighted genes of T cell exhaustion model. **B**. Enrichment analysis on the top 50 positively/negatively weighted genes of T cell exhaustion model. **C**. Top 20 positively/negatively weighted genes of EMT model. **D**. Enrichment analysis on the top 50 positively/negatively weighted genes of EMT model. **E**. Top 20 positively/negatively weighted genes of hypoxia model. **F**. Enrichment analysis on the top 50 positively/negatively weighted genes of hypoxia model. **G**. Top 20 positively/negatively weighted genes of cell cycle model. **H**. Enrichment analysis on the top 50 positively/negatively weighted genes of cell cycle model. Negative counts indicate enrichments for negatively weighted genes and the positive counts indicate positively weighted ones.

For the T cell exhaustion model trained by the Neal et al. dataset [30], the canonical marker genes such as *PDCD1*, *LAG3*, and *HAVCR2* showed high-rank weights with no surprise (**Figure 3**A). The enrichment analysis based on the top positively weighted genes of T cell exhaustion hit the “regulation of cell activation” (Figure 3B), while the enrichment analysis based on the top negatively weighted genes were enriched for “lymphocyte activation”. Obviously, they were two polarized states of T cells.

For the EMT model trained by the Lin et al. dataset [32], the top positively weighted genes included *SERPINE1*, *VIM*, *SNAI2*, and *ANGPTL4*, which were also expected (Figure 3C). The top positively weighted genes exactly hit the “hallmark of EMT” (Figure 3D). The top negatively weighted genes of EMT hit the “endopeptidase activity”, which has never been discussed and probably needs extra attention.

For the hypoxia model trained by the Darmanis et al. dataset [34], *VEGFA*, *CAV1*, and *CA9* emerged at the top positively weighted genes (Figure 3E), which were canonical hypoxia-related genes. The top positively weighted genes exactly hit the “hallmark of hypoxia”, which again validated the reliability of our framework (Figure 3F**)**. Interestingly, the enrichment analysis based on the top positively weighted genes of hypoxia also hit the “hallmark of glycolysis”. This correlation has been reported in a previous study [38]. Such correlations, as byproducts, won’t be naturally investigated when employing a canonical gene set-based scoring system to evaluate the cellular state.

For the cell cycle model trained by the Zhang et al. dataset [36], *MKI67*, *STMN1*, *MCM4*, and *TOP2A* showed up at the top positively weighted genes (Figure 3G), which were canonical cell cycle-related genes. These top positively weighted genes hit the terms such as “cell division” and “cell cycle checkpoint” (Figure 3H).

In summary, the top positively weighted genes are mostly involved in the pathways that drive the development of the corresponding cellular statuses/features, and the top negatively weighted genes are involved in signature pathways of the opposite cellular states. It makes the ANN-based model parameters interpretable.

Other than the canonical genes that have been included in the corresponding enriched terms, there are some highly weighted genes that have been mentioned in previous literature, suggesting that their roles in the cells exhibiting the corresponding features are corroborated by two independent studies, which is non-trivial. These genes are listed in Table S3. Notably, some of the genes with the top weights have not been previously reported in the literature. While these may represent artifacts or confounders, they hold the potential to be as significant as those canonical markers and those documented in Table S3. For comprehensive scrutiny, we listed both positively and negatively weighted genes associated with the trained features in Table S4, available for any researchers inclined toward further exploration.

### SuperFeat outperforms gene set-based scoring methods in discerning the target subpopulations

**Figure 4:**
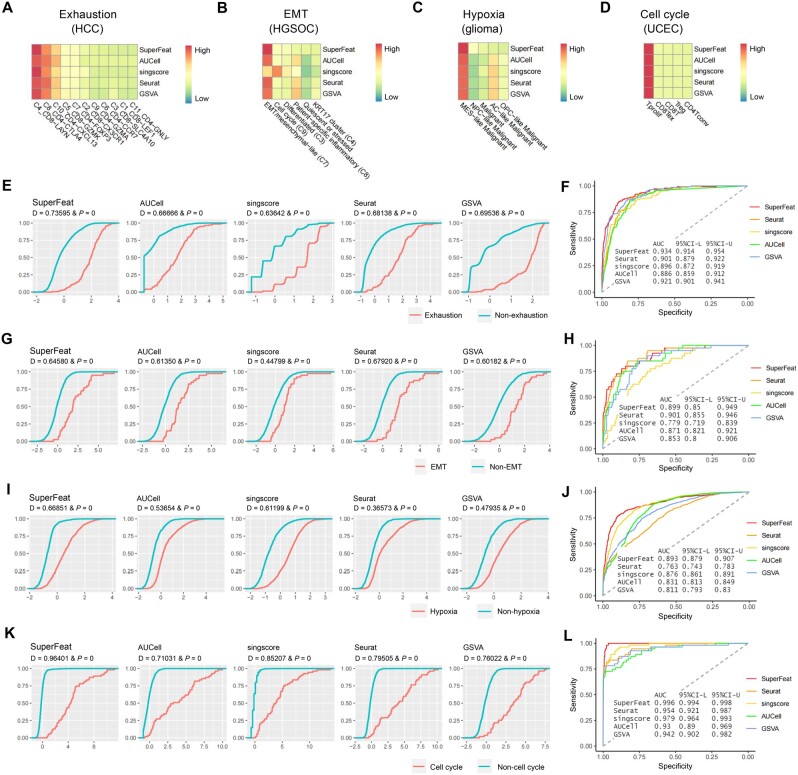
Comparison of cellular status/feature scoring methods Heatmaps for median signal scores evaluated by SuperFeat, AUCell, singscore, Seurat, and GSVA for T cell exhaustion (**A**), EMT (**B**), hypoxia (**C**), and cell cycle (**D**). **E**. K-S statistics on T cell exhaustion state. **F**. ROC analysis on T cell exhaustion state. **G**. K-S statistics on EMT state. **H**. ROC analysis on EMT state. **I**. K-S statistics on hypoxia state. **J**. ROC analysis on hypoxia state. **K**. K-S statistics on cell cycle state. **L**. ROC analysis on cell cycle state. K-S, Kolmogorov-Smirnov; ROC, receiver operator characteristic curve; AUC, area under curve; GSVA, gene set variation analysis; CI, confidence interval; CI-L, the lower bound of CI; CI-U, the upper bound of CI.

Prior to SuperFeat, people oftentimes used the gene set-based scoring methods, such as Seurat modular score [21,39,40], AUCell [22], singscore [23], and GSVA [24], to evaluate the cellular state based on a group of canonical genes. To efficiently discern the cell subpopulations of a certain cell type that reflect the variability of a certain cellular status/feature, the separation of the signal distributions of the scores suggests a better performance. As shown in the heatmaps of median signal scores evaluated by SuperFeat, Seurat, AUCell, singscore, and GSVA in the four validation datasets (**Figure 4**A–D), it appeared that SuperFeat scores made the target subpopulation stand out with better color contrast than others. We further used the Kolmogorov-Smirnov (K-S) statistics to numerically evaluate how much the subpopulations with certain cellular statuses/features stand out from their counterpart subpopulations without this feature. The results showed that SuperFeat mostly outperformed other gene set-based scoring systems by the relatively larger K-S D values and larger area under curves (AUCs) (Figure 4E–L), except being slightly lower than the Seurat scores for EMT gene set. More detailed results of the accuracy analysis are shown in Table S5. Canonical marker genes for all the specific cellular states are listed in Table S6.

Overall, it is confident to claim that SuperFeat discerns the clusters annotated with the designated cellular statuses/features more easily and accurately than other gene set-based scoring methods. Furthermore, the advantage of SuperFeat scoring resides in its independency of the canonical gene set, which saves the arbitrariness of gene selection.

In addition, SuperFeat also showed comparable performance with logistic regression (Figure S3). However, SuperFeat exhibited superior universality and flexibility as a neural network model and offered variability such as the number of hidden layers and connection structures, providing more possibilities for future efficient and artificial general intelligence.

### SuperFeat scoring in ST study

**Figure 5:**
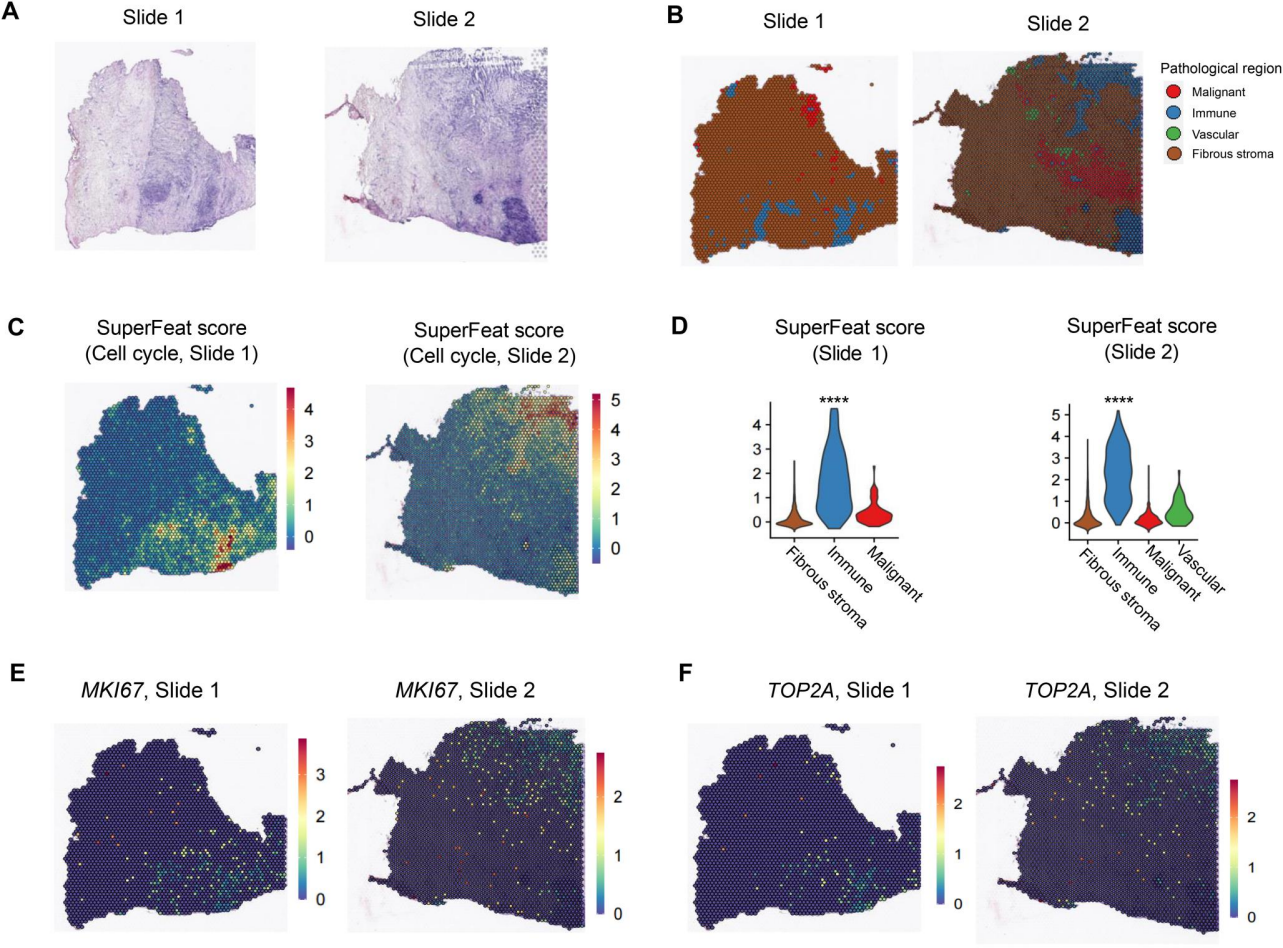
SuperFeat scoring on two ST slides **A**. H&E staining for two slides from the same tumor tissue. **B**. Histological annotation by the pathologist. **C**. SuperFeat cell cycle scores of the dots on the two replicate slides of 10X Genomics Visium platform. **D**. Violin plots of SuperFeat cell cycle scores of the dots on the two replicate slides. **E**. *MKI67* signals of the dots on the two replicate slides. **F**. *TOP2A* signals of the dots on the two replicate slides. The significance was determined by Wilcoxon test (****, *P* < 0.0001). ST, spatial transcriptomics; H&E, hematoxylin and eosin.

The SuperFeat scoring model can also be applied to the ST data, which allows us to correlate the cellular statuses/features to the histology. We used the two 10X Genomics Visium slides from the tumor samples of a cholangiocarcinoma cancer patient to demonstrate the mapping of the proliferative signals. **Figure 5**A shows the original H&E staining of the two slides from the same tumor tissue. Figure 5B shows the annotation of the pathological regions of the two slides determined by an experienced pathologist. Figure 5C shows the landscape of the proliferative signals on the two slides. Figure 5D shows the violin plots of the proliferative SuperFeat scores of the two slides. From the two replicate slides of the same tissue, the high cell cycle signal was reproducibly enriched in the immune regions, suggesting the high proliferative potential. We also mapped the signals of the two signature genes, *MKI67* and *TOP2A*, on the 10X Genomics Visium slides (Figure 5E and F). The landscape of the two genes’ signals appeared more stochastic than the SuperFeat proliferation signal, suggesting the advantage of a comprehensive evaluation. The other three tumor-related feature scores are shown in Figure S4.

### Repurposing drug search using SuperFeat model parameters

As the malignant cellular states such as tumor proliferation and EMT could be targeted for cancer therapy, similar to CMap strategy, we were able to perform the search based on the gene weights in the trained cellular state model against the perturbagen databases such as CMap and LINCS L1000. Without confounding the variability derived from the heterogeneous composition of the cell populations in the samples, we intuitively believed that the drug search using the top weight-ranked genes derived from single-cell data could be more specific to the cellular state change than the differential genes found in bulk RNA transcriptomic data but more general in the application targeting the detrimental cellular state.

Using the CMap search strategy over the LINCS L1000 data repositories implemented by the signatureSearch package, we tested such an idea and got interesting results.

**Figure 6:**
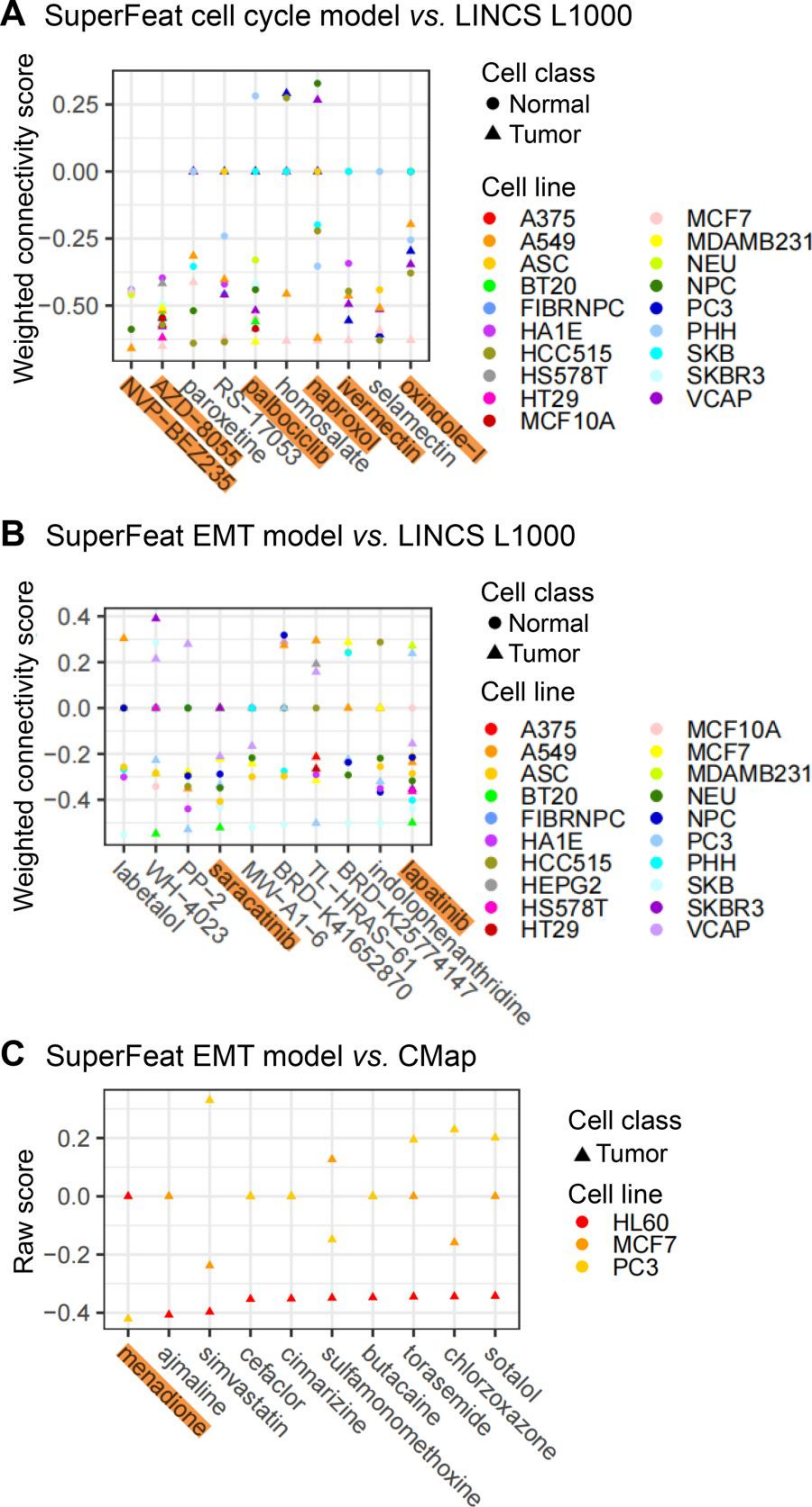
Drug search using weight-ranked genes of SuperFeat **A**. Drug search result based on the top positively/negatively weighted genes of the SuperFeat cell cycle model and the LINCS L1000 database. **B**. Drug search results based on the top positively/negatively weighted genes of the SuperFeat EMT model and the LINCS L1000 database. **C**. Drug search results based on the top positively/negatively weighted genes of the SuperFeat EMT model and the CMap database. CMap, the Connectivity Map.

**Figure 6**A shows the drug search based on the top weighted genes derived from the cell cycle features. Among the top 10 hits on LINCS L1000, AZD8055 [41], palbociclib [42], NVP-BEZ235 [43], naproxol [44], ivermectin [45], and oxindole-I [46] are the cell cycle arrest agents that have been previously reported.

The drug search based on the top weighted genes derived from the EMT features also gives meaningful results. Among the top 10 hits on LINCS L1000, saracatinib has been reported to be involved in the restoration of E-cadherin expression [47], and lapatinib plays a role in EMT [48] (Figure 6B). The top 1 hit on CMap, menadione (vitamin K3) has been reported to be involved in suppression of EMT [49] (Figure 6C).

### Stability of model parameters and reproducibility of SuperFeat results

As the model parameters are the major determinants of the drug search, in order to evaluate the robustness of the methods, we compared the top weighted genes and the output drugs derived from different training datasets using the most established cellular feature, *i.e.*, proliferation.

**Figure 7:**
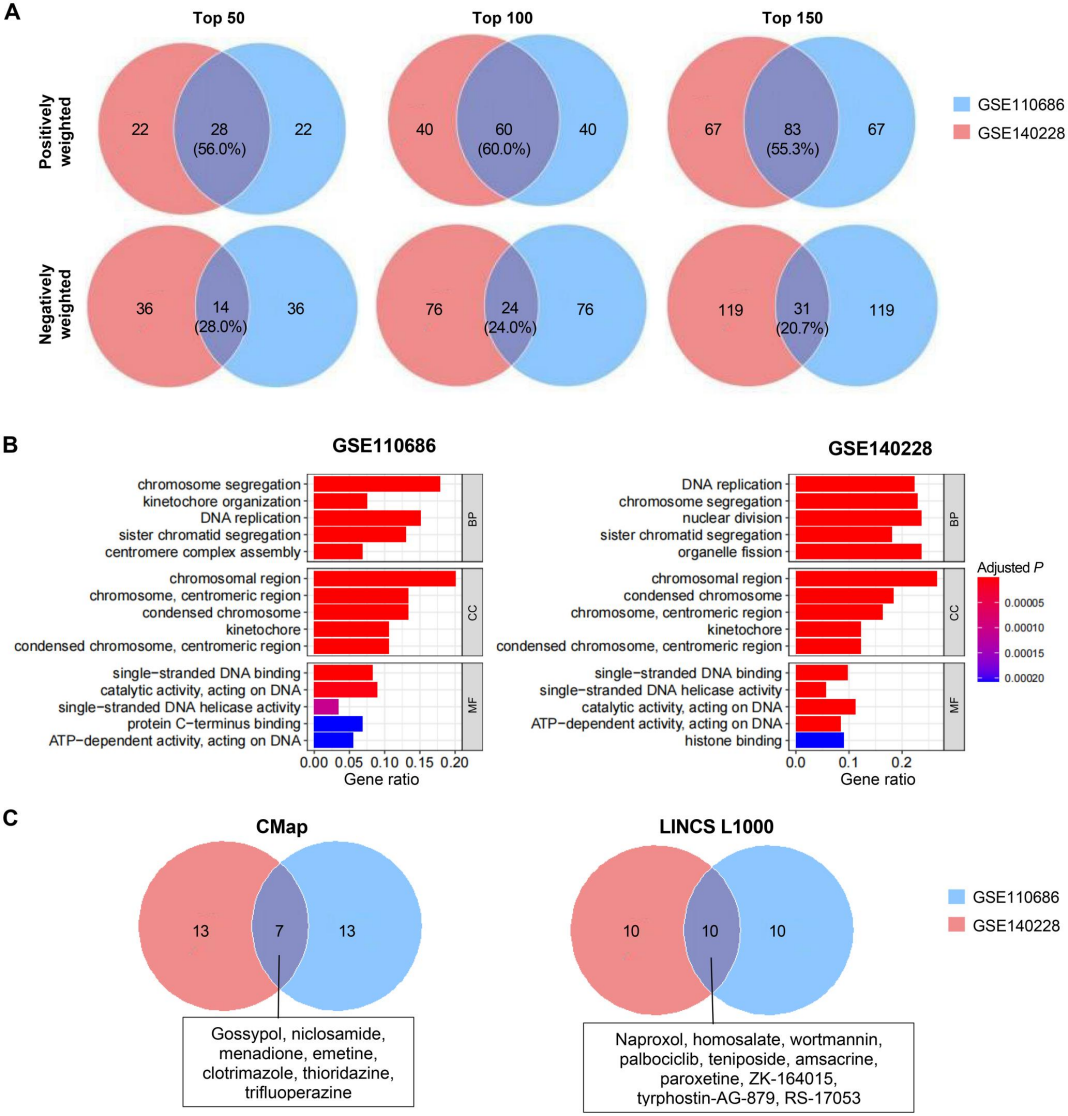
Stability of model parameters and reproducibility of SuperFeat results of cell cycle state/feature **A**. Venn diagrams showing stable weighted genes between different datasets. The number indicates gene count. **B**. Bar plots displaying similar GO terms. Only top 5 categories are shown here. **C**. Venn diagrams showing the drugs repeatedly searched in CMap and LINCS L1000. The detailed drugs are listed in the boxes. The number indicates drug count. GO, Gene Ontology; BP, biological process; CC, cellular component; MF, molecular function.

The datasets were retrieved from GEO databases: GSE140228 for HCC proliferation and GSE110686 for T cell proliferation. Although different cell types were involved in the same cellular state, our results showed that 55%–60% of the top positively weighted genes were reproducible in two datasets (**Figure 7**A). There were also 21%–28% of negatively weighted genes overlapped, whose roles remain elusive. The enrichment analysis of the top positively weighted genes mostly hit the same Gene Ontology (GO) terms (Figure 7B). At last, we compared the output drugs. For the CMap search, 7 out of 20 top selections were reproducible. For the LINCS L1000 search, 10 out of 20 top selections were reproducible (Figure 7C). It was also found that naproxol and palbociclib were always among the top selections of both datasets in the LINCS L1000 search.

Such reproducible results suggest that, despite the training datasets being different, similar genes that contribute to the canonical cellular statuses/features will be assigned high weights. These genes will also subsequently provide the leads necessary to discover drug candidates that either promote beneficial cellular states or inhibit adverse ones.

Figures S5 and S6 display additional results that assess the stability of the features for hypoxia and T cell exhaustion, respectively. The overlap of high-weight genes remains significant for both features, underscoring the robustness of our findings. However, the limited number of cells in some published datasets could restrict our validation of reproducibility. We were unable to identify a sufficient number of cells to extend our reproducibility validation further, such as EMT. This limitation is evidenced in Table S1, which illustrates that the state1 cell populations in certain datasets are too small to train a robust model. Such dataset could still be used for score testing though.

### *In vitro* and *in vivo* validation of drug effects on adverse cellular state development in tumor

In a collaborative study of the cancer-associated fibroblasts (CAFs), we discovered a state transition of CAFs in a subcutaneous melanoma mouse model. The dataset included 101,684 cells from the tumor tissue. It allows to delineate nine distinct tumor micro-environment (TME) types: T cells, B cells, dendritic cells, neutrophils, macrophages, fibroblasts, endothelial cells, melanoma cells, and stem/progenitor-like cells. Focusing on CAFs, our analysis revealed a novel subpopulation marked by CD34^+^PI16^+^, which gave rise to the classical ACTA2^+^MCAM^+^ CAFs in tumor, also known as myCAF (data unpublished). The lineage connection of these CAFs with these markers were confirmed using tdTomato marker.

**Figure 8:**
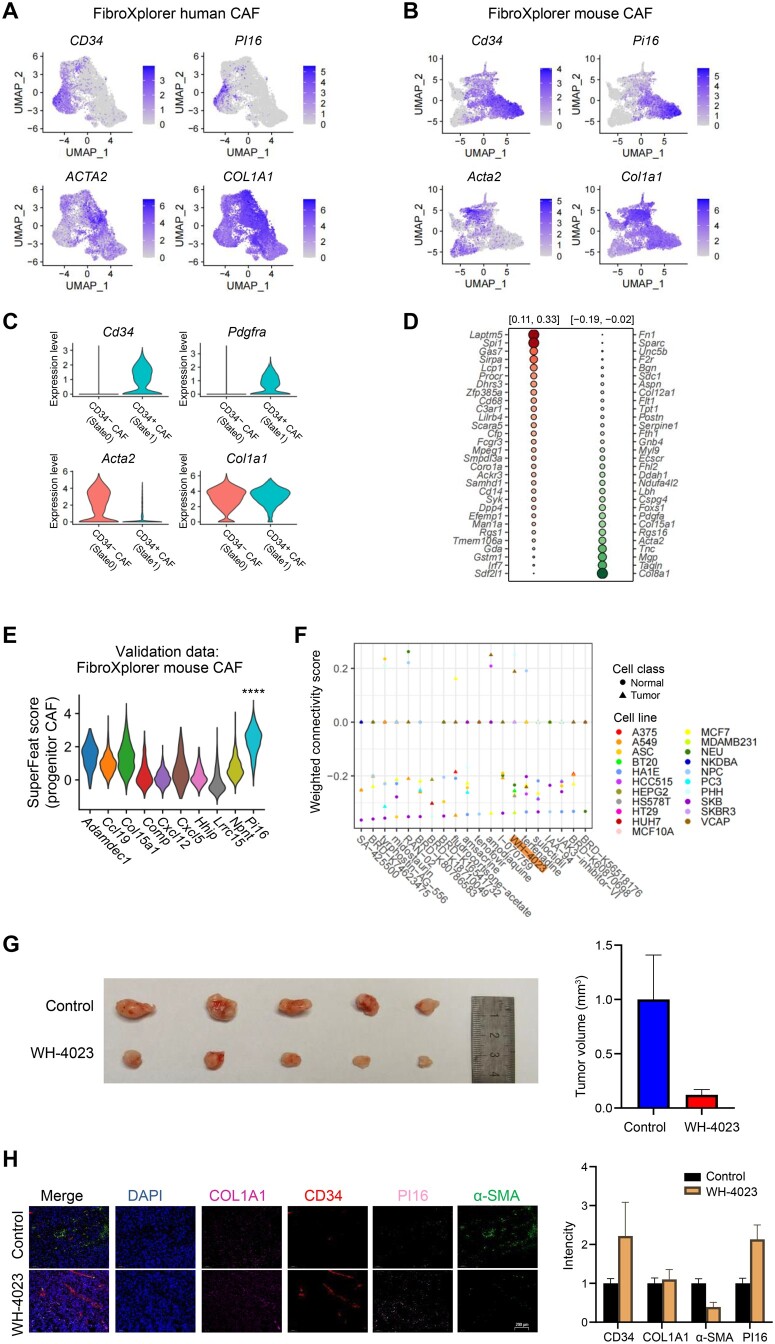
*In vitro* and *in vivo* validation of a drug candidate by SuperFeat and signatureSearch **A**. Fibroblast subpopulations in human tissues from FibroXplorer database. **B**. Fibroblast subpopulations in mouse tissues from FibroXplorer database. **C**. Violin plot for the fibroblast subpopulations in our CAF development study in subcutaneous tumor mice. **D**. Top 30 positively/negatively weighted genes for CAF development SuperFeat model. **E**. Validation of CAF development SuperFeat model using FibroXplorer mouse dataset. The significance was determined by Wilcoxon test (****, *P* < 0.001). **F**. Top 20 drug search candidates. **G**. The effect of WH-4023 on tumor shrinkage. **H**. The effect of WH-4023 on fibroblast development using immunofluorescence staining. Scale bar, 200 μm. FibroXplorer is available at https://www.fibroxplorer.com/home. UMAP, Uniform Manifold Approximation and Projection; CAF, cancer-associated fibroblast.

Similar CAF subpopulations were characterized in both human and mouse tissues in the published datasets [50] (**Figure 8**A and B). The developmental trajectory of CAFs from the CD34^+^ fibroblast progenitor state to the ACTA2^+^ classical CAF state was defined and validated in our collaborative study. State1 (CD34^+^ CAF) represents a more stemness state characterized by the signature genes such as *Cd34* and *Pdgfra*. State0 (Cd34^−^ CAF) represents a more differentiation state with the tumor-prone signature marker such as *Acta2* (Figure 8C).

Using SuperFeat framework, we were hence able to train a new stemness scoring model to evaluate the progression of CAF differentiation in a tumoral biopsy. A total of 4594 cells which were composed of CD34^+^PI16^+^ progenitor cells and ACTA2^+^MCAM^+^ myCAF cells were used for training (Table S1).

Using the subsequent search, we aimed to find the drugs that potentially suppress the conversion from the progenitor State1 to the tumor-prone State0 (Figure 8D and E). The top 20 drug candidates from LINCS L1000 are shown in Figure 8F. By validation experiments on these 20 candidates, four of them showed very promising outcomes. Figure 8G shows the outcome of MKN-45 (gastric cancer cell) subcutaneous tumor model with the treatment of WH-4023, which is one of the four drugs. The tumors significantly shrank in five replicates.

To confirm that the drug indeed inhibits tumor growth via the suppression of CAF development, we performed immunofluorescence staining of gastric subcutaneous tumors for signature markers, including CD34 and PI16 for the progenitor state, and ACTA2 (also known as α-SMA) and COL1A1 for the classic tumor-prone state. Notably, the WH-4023 treatment group exhibited lower α-SMA signals but higher CD34 and PI16 signals in comparison with the control group (Figure 8H).

## Discussion

In this study, we have established and demonstrated an innovative framework designed to assess cellular states, tissue development, and even individual patient conditions. This framework comprises a few critical elements: (1) an R package that facilitates the evaluation of canonical cellular states via a scoring system; (2) a data repository containing model parameters of various documented cellular states from previous research; and (3) Python training code for user-defined cellular states. This setup allows for the efficient evaluation of canonical cellular states previously identified in research. Users can leverage this framework to simply assess cellular states using existing models, or train their own SuperFeat scoring models, and potentially propagate their own cellular state models.

We have specifically demonstrated how the SuperFeat model assesses several key cellular states or features of tumors, such as cell proliferation, T cell exhaustion, hypoxia, and EMT, all of which are hallmark cancer signals. These examples underscore the simplicity, generalizability, and efficiency of the new method. We strived to demystify the “black box” of the SuperFeat ANN model by visualizing and interpreting the model parameters, specifically the gene weights potentially contributing to a specific cellular state. Compared to the signature gene set-based scoring methods, SuperFeat demonstrates superior performance because it considers not only the up-regulation of signature genes, but also suppressed genes. Such contributions are reflected by positive and negative weights, respectively. By examining the weights of genes, researchers may acquire additional insights.

Essentially, SuperFeat provides additional learning framework and a scoring strategy that streamline the rapid assessment of states or features in tissue, many of which have been extensively explored and acknowledged in existing literature. Moreover, compared with other traditional methods, SuperFeat renders such assessment more intuitive, generalizable, and user-friendly, without relying on the details of the gene set. While an ANN model bypasses the uncertainties of statistical assumptions in a rigorous mathematical model, the weight parameters of genes derived from such a training still align remarkably well with expectations. Logically, the metric values of the SuperFeat cellular status/feature reflect the resemblance between the putative cells of interest in the training dataset and the cells in the new study, in comparison to their counterparts. Therefore, the model’s reliability largely depends on the quality of the training dataset. As our understanding of cells and single-cell transcriptomics datasets keep growing, the SuperFeat framework is set to make the automated learning and assessment of cellular statuses/features increasingly reliable and efficient.

An even more thrilling advantage of this framework lies in its potential for downstream applications, *e.g.*, repurposing drug searches. The other gene set-based approaches of cellular state evaluation are not comparable in this aspect. As we know, CMap was based on the connectivity between gene expression profiles and drug perturbations. Previous CMap perturbation datasets were derived from bulk RNA-seq. The recent progress of the scRNA-seq technique makes it practical to generate such connectivity at single-cell resolutions. The advantage of our strategy could heighten the precision and success rate of expression profile-based drug searches. We successfully conducted the validation experiments that substantiate the feasibility of such strategy. Another advantage of neural network-based strategy pertains to the convenience of the interconnection with other models. It makes integration of drug search tasks based on multiple data sources possible.

In brief, our effort aims to build a preliminary and versatile framework that allows for the rapid and automatic evaluation of the conditions of multicellular samples by investigating single-cell transcriptomics, eventually finding solutions to more complex biological and clinical problems. ANN-based SuperFeat architecture not only can be used independently, but also enables us to generate small network modules that can be assembled and integrated. We have shown results suggesting high consistency between the single-node SuperFeat model and logistic regression, which makes SuperFeat model interpretable. However, considering the integrability of multiple models, an ANN-based model offers greater versatility and compatibility to build a more complex network structure and to accomplish more intricate learning tasks, especially when the current machine learning programming architecture is predominantly based on Python libraries such as TensorFlow, PyTorch, or Keras. More interesting learning strategy can therefore be realized. For example, the pre-trained SuperCT and SuperFeat models can be easily assembled into a deeper learning network model that can be further trained for new tasks in a transfer learning fashion (by “freezing” pre-trained parameter sub-networks in new model training). Such a deeper network model enables the solving of more complex problems involving cell type classification and feature quantitation with better interpretability of SuperCT and SuperFeat components. We anticipate further developments in the biological field driven by the efforts of AI scientists with more careful consideration of the benefits from SuperFeat and SuperCT components in single-cell transcriptomics studies.

## Ethical statement

All animal breeding, housing, and experimentation were conducted according to the guidelines of the Institutional Committee of Shanghai Jiao Tong University School of Medicine for Animal Research, China (Approval No. XHEC-F-2020-026).

## Code availability

The framework is implemented in R package rSuperFeat and can be accessed at https://github.com/weilin-genomics/rSuperFeat.

## CRediT author statement

**Jianmei Zhong:** Methodology, Investigation, Visualization, Writing – review & editing. **Junyao Yang:** Investigation, Visualization, Writing – review & editing. **Yinghui Song:** Investigation, Visualization. **Zhihua Zhang:** Investigation, Visualization. **Chunming Wang:** Investigation. **Renyang Tong:** Investigation. **Chenglong Li:** Investigation. **Nanhui Yu:** Investigation. **Lianhong Zou:** Investigation. **Sulai Liu:** Investigation. **Jun Pu:** Supervision, Writing – review & editing. **Wei Lin:** Conceptualization, Methodology, Supervision, Writing – original draft, Writing – review & editing. All authors have read and approved the final manuscript.

## Supplementary material

Supplementary material is available at *Genomics, Proteomics & Bioinformatics* online (https://doi.org/10.1093/gpbjnl/qzae036).

## Competing interests

The authors have declared no competing interests.

## Supplementary Material

qzae036_Supplementary_Data
