# Supplementary material for: SuperFeat: Quantitative Feature Learning from Single-cell RNA-seq Data Facilitates Drug Repurposing
Source: Genomics Proteomics Bioinformatics. 2024 May 23;22(3):qzae036. doi: 10.1093/gpbjnl/qzae036 (PMC12016572; doi:10.1093/gpbjnl/qzae036)
Supplement: qzae036_Supplementary_Data [file qzae036_supplementary_data.zip › Table S5-done.docx]

**Table S5 Outperformance of SuperFeat over gene-set-based scoring in discerning the target subpopulations**

| Exhaustion | AUC | 95%CI-L | 95%CI-U | Specificity | Sensitivity | Accuracy |
| --- | --- | --- | --- | --- | --- | --- |
| SuperFeat | 0.934 | 0.914 | 0.954 | 0.885 | 0.851 | 0.883 |
| Seurat | 0.901 | 0.879 | 0.922 | 0.809 | 0.872 | 0.814 |
| singscore | 0.896 | 0.872 | 0.919 | 0.863 | 0.773 | 0.857 |
| AUCell | 0.886 | 0.859 | 0.912 | 0.808 | 0.858 | 0.812 |
| GSVA | 0.921 | 0.901 | 0.941 | 0.823 | 0.872 | 0.827 |
| EMT |  |  |  |  |  |  |
| SuperFeat | 0.899 | 0.850 | 0.949 | 0.871 | 0.775 | 0.868 |
| Seurat | 0.901 | 0.855 | 0.946 | 0.829 | 0.850 | 0.830 |
| singscore | 0.779 | 0.719 | 0.839 | 0.673 | 0.775 | 0.676 |
| AUCell | 0.871 | 0.821 | 0.921 | 0.764 | 0.850 | 0.766 |
| GSVA | 0.853 | 0.800 | 0.906 | 0.752 | 0.850 | 0.755 |
| Hypoxia |  |  |  |  |  |  |
| SuperFeat | 0.893 | 0.879 | 0.907 | 0.884 | 0.784 | 0.831 |
| Seurat | 0.763 | 0.743 | 0.783 | 0.561 | 0.805 | 0.692 |
| singscore | 0.876 | 0.861 | 0.891 | 0.786 | 0.826 | 0.808 |
| AUCell | 0.831 | 0.813 | 0.849 | 0.652 | 0.885 | 0.777 |
| GSVA | 0.811 | 0.793 | 0.830 | 0.729 | 0.751 | 0.741 |
| Cell cycle |  |  |  |  |  |  |
| SuperFeat | 0.996 | 0.994 | 0.998 | 0.964 | 1.000 | 0.965 |
| Seurat | 0.954 | 0.921 | 0.987 | 0.977 | 0.818 | 0.973 |
| singscore | 0.979 | 0.964 | 0.993 | 0.888 | 0.964 | 0.891 |
| AUCell | 0.930 | 0.890 | 0.969 | 0.983 | 0.727 | 0.976 |
| GSVA | 0.942 | 0.902 | 0.982 | 0.978 | 0.782 | 0.973 |

*Notes*: The 95% CI was computed with 2000 stratified bootstrap replicates using pROC package. CI-L indicates the lower bound of the CI and CI-U indicates the upper bound of the CI. AUC, area under curve; CI, confidence interval; EMT, epithelial–mesenchymal transition; GSVA, gene set variation analysis.
