# Supplementary material for: SuperFeat: Quantitative Feature Learning from Single-cell RNA-seq Data Facilitates Drug Repurposing
Source: Genomics Proteomics Bioinformatics. 2024 May 23;22(3):qzae036. doi: 10.1093/gpbjnl/qzae036 (PMC12016572; doi:10.1093/gpbjnl/qzae036)

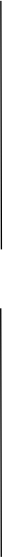


**A**

**B**

SuperFeat score

(Exhasution)


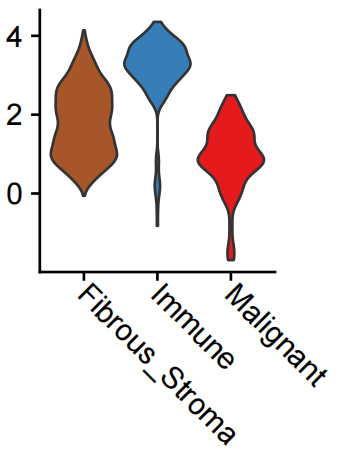

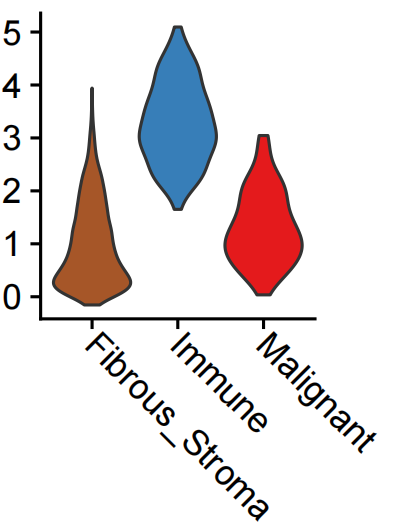

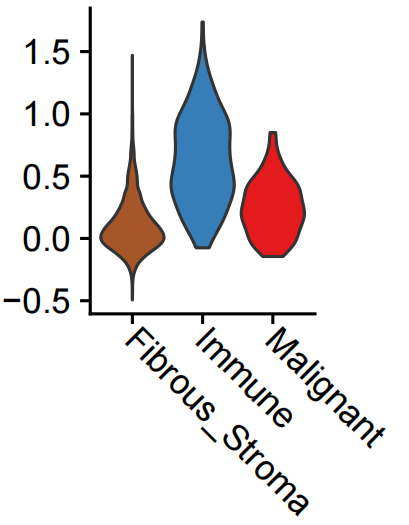


SuperFeat score

(Exhasution)


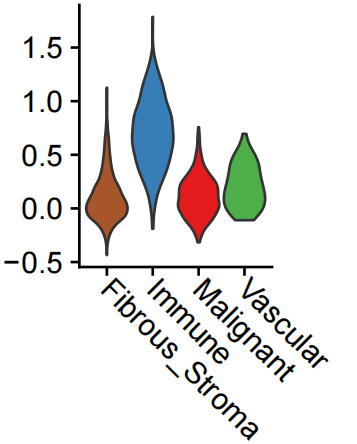


SuperFeat score (EMT)

SuperFeat score (EMT)


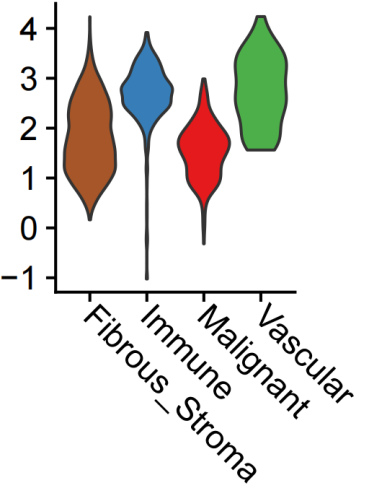


SuperFeat score

(Hypoxia)

**Slide 1 Slide 2**

SuperFeat score

(Hypoxia)


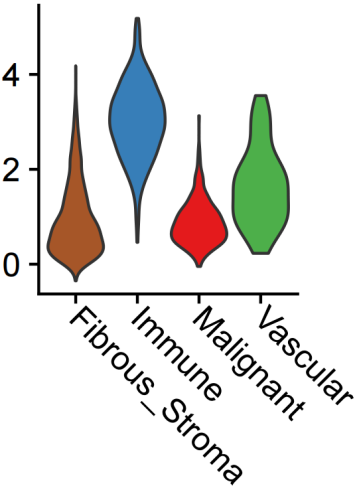

Supplement: qzae036_Supplementary_Data [file qzae036_supplementary_data.zip › Figure S4-done.docx]
