# Supplementary material for: SuperFeat: Quantitative Feature Learning from Single-cell RNA-seq Data Facilitates Drug Repurposing
Source: Genomics Proteomics Bioinformatics. 2024 May 23;22(3):qzae036. doi: 10.1093/gpbjnl/qzae036 (PMC12016572; doi:10.1093/gpbjnl/qzae036)
Supplement: qzae036_Supplementary_Data [file qzae036_supplementary_data.zip › Supplementary material captions.docx]

**Supplementary material**

**Figure S1 UMAP layout of the training dataset for T cell exhaustion model**

The dashed circle indicates the state1 cell subpopulation.

**Figure S2 T cell annotations in validation datasets**

*t*-SNE layout of T cell exhaustion in HCC validation dataset. *t*-SNE, *t*-distributed stochastic neighbor embedding.

**Figure S3 SuperFeat feature scores based on the spatial transcriptomics**

**A.** Violin plots for T cell exhaustion, EMT, and hypoxia signals on slide 1. **B.** Violin plots for T cell exhaustion, EMT, and hypoxia signals on slide 2.

**Figure S4 Stability of model parameters and reproducibility of T cell exhaustion**

**A.** Venn diagrams show the intersection of weighted genes. Top panel is positively weighted genes and bottom panel is negatively weighted genes. The number indicates genes count. **B.** The barplots display similar GO terms. Only top 5 categories are shown.

**Figure S5 Stability of model parameters and reproducibility of hypoxia**

**A.** Venn diagrams show the intersection of weighted genes. Top panel is positively weighted genes and bottom panel is negatively weighted genes. The number indicates genes count. **B.** The barplots display similar GO terms. Only top 5 categories are shown.

**Figure S6 Comparison of performance between SuperFeat and linear regression**

**A.** Scores on exhaustion state in the train and validation datasets. Left panel is violin plots and ROC curves of scores of KIRC cell subpopulation; right panel is violin plots and ROC curves of scores of NSCLC cell subpopulation. **B.** Scores on EMT state in the train and validation datasets. Left panel is violin plots and ROC curves of scores of PDAC cell subpopulation; right panel is violin plots and ROC curves of scores of HGSOC cell subpopulation. **C.** Scores on cell cycle state in the train and validation datasets. Left panel is violin plots and ROC curves of scores of LIHC cell subpopulation; right panel is violin plots and ROC curves of scores of UCEC cell subpopulation. **D.** Scores on hypoxia state in the train and validation datasets. Left panel is violin plots and ROC curves of scores of glioma cell subpopulation from GSE84465; right panel is violin plots and ROC curves of scores of glioma cell subpopulation from GSE131928. The red boxes highlight the target cell subpopulation. NSCLC, non-small cell lung cancer; LR, linear regression.

**Table S1 The datasets for feature training**

**Table S2 19,202 input features for training**

**Table S3 Genes not included in top10 enrichment terms but have significant association with the trained cell states in previous literature**

**Table S4 Feature weights for each cell state**

**Table S5 Outperformance of SuperFeat over gene-set-based scoring in discerning the target subpopulations**

**Table S6 Canonical markers used by** **AddModuleScore function in Seurat package for comparison among different scoring methods**
