# Supplementary material for: SuperFeat: Quantitative Feature Learning from Single-cell RNA-seq Data Facilitates Drug Repurposing
Source: Genomics Proteomics Bioinformatics. 2024 May 23;22(3):qzae036. doi: 10.1093/gpbjnl/qzae036 (PMC12016572; doi:10.1093/gpbjnl/qzae036)

**A** **Train: KIRC Validation: NSCLC**

**Validation: HGSOC**


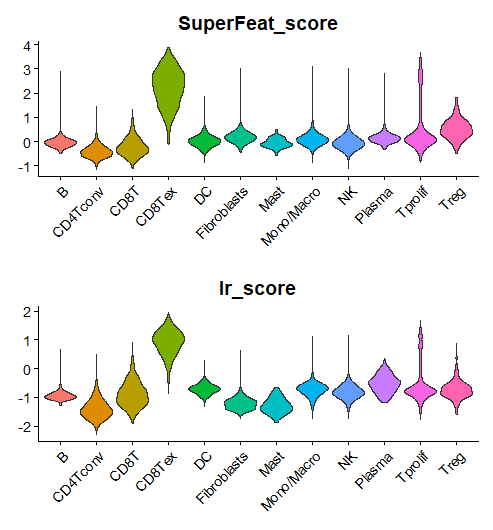

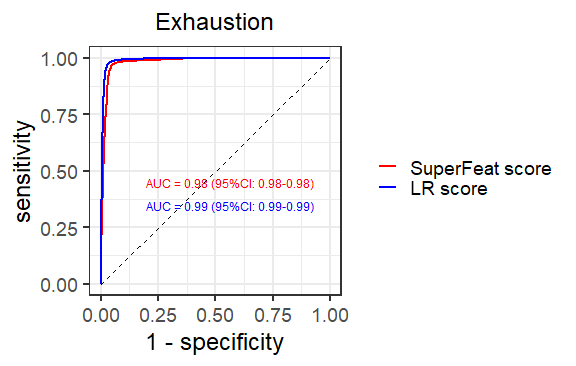

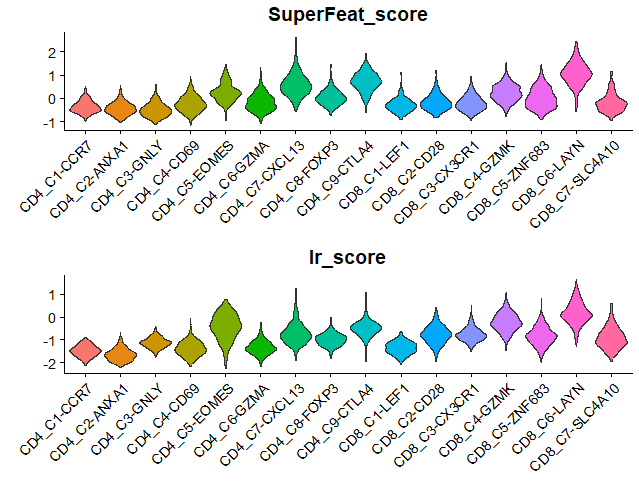

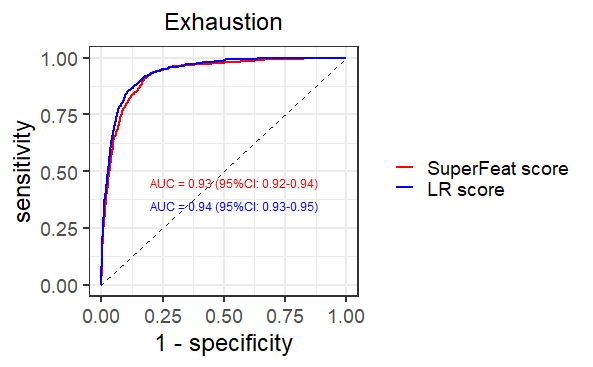

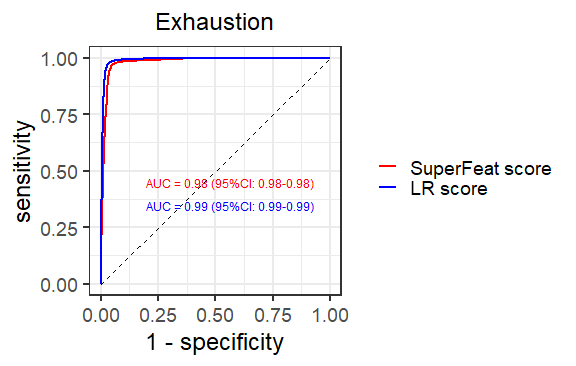


**Train: LIHC**

**C**

**Validation: UCEC**

**Train: PDAC**

**B**


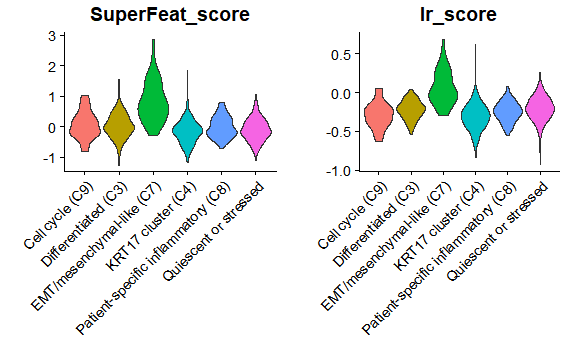

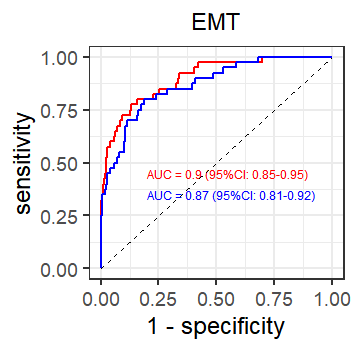

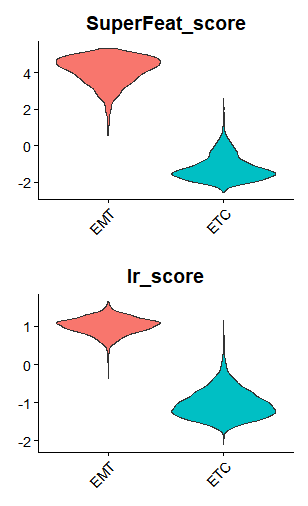

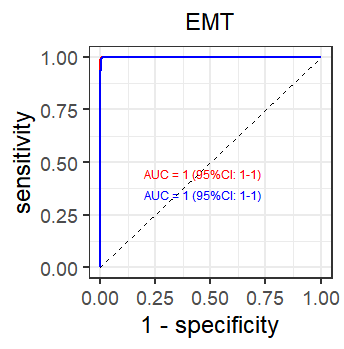


Cell cycle


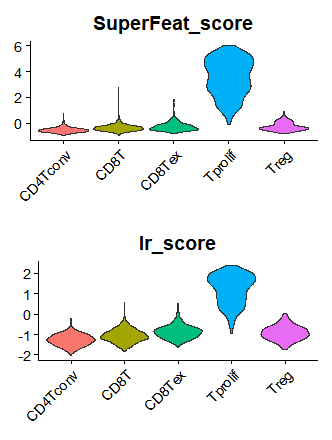

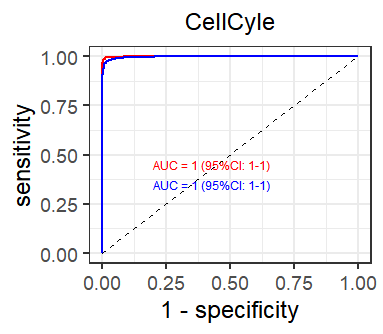


**D Train: glioma GSE84465**


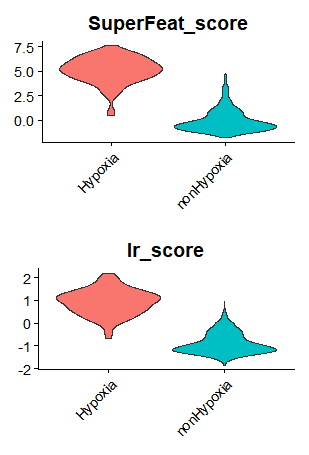

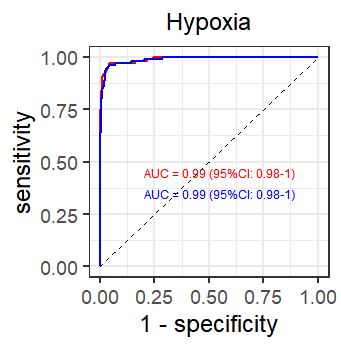


Cell cycle


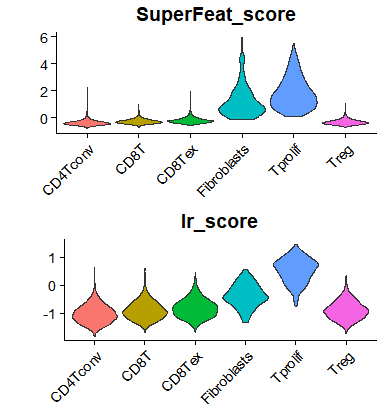

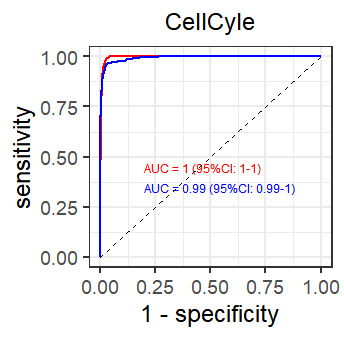


**Validation: glioma GSE131928**


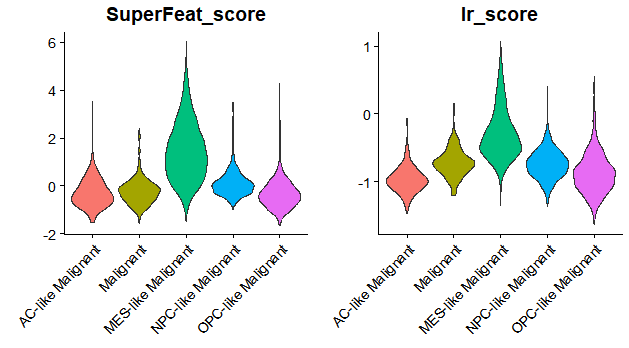

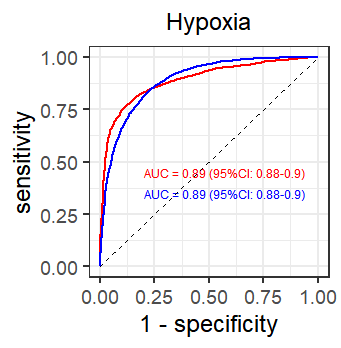

Supplement: qzae036_Supplementary_Data [file qzae036_supplementary_data.zip › Figure S3-done.docx]
