# Supplementary material for: SuperFeat: Quantitative Feature Learning from Single-cell RNA-seq Data Facilitates Drug Repurposing
Source: Genomics Proteomics Bioinformatics. 2024 May 23;22(3):qzae036. doi: 10.1093/gpbjnl/qzae036 (PMC12016572; doi:10.1093/gpbjnl/qzae036)
Supplement: qzae036_Supplementary_Data [file qzae036_supplementary_data.zip › Table S1-done.docx]

**Table S1 The datasets for feature training**

| **State** | **Type** | **Dataset** | **Accession** | **Cell number** | **Cell type** | **State1** | **State0** |
| --- | --- | --- | --- | --- | --- | --- | --- |
| Exhaustion | Train | KIRC | GSE111360 [1] | 2777 | CD8 T cells | 1532 | 1245 |
| Exhaustion | Validation | HCC | GSE98638 [2] | 3636 | T cells | 250 | 3386 |
| Exhaustion | Validation | NSCLC | GSE99254 [3] | 9055 | T cells | 493 | 8562 |
| EMT | Train | PDAC | GSE154778 [4] | 4604 | Epithelial cells | 989 | 3615 |
| EMT | Validation | HGSOC | GSE132149 [5] | 1410 | Epithelial cells | 40 | 1370 |
| Hypoxia | Train | Glioma | GSE84465 [6] | 915 | Neoplastic | 108 | 807 |
| Hypoxia | Validation | Glioma | GSE131928 [7] | 7062 | Malignant | 3877 | 3185 |
| Cell cycle | Train | LIHC | GSE140228 [8] | 3020 | T cells | 1020 | 2000 |
| Cell cycle | Validation | UCEC | GSE139555 [9] | 12,692 | T cells | 284 | 12,408 |
| Cell cycle | Validation | BRCA | GSE110686 [10] | 804 | T cells | 304 | 500 |
| Quiescent | Train | CRC | GSE163974 [11] | 218 | Cancer stem cells | 45 | 173 |
| Differentiation | Train | LUNG | GSE135893 [12] | 16,033 | Ciliated cells | 1362 | 14,671 |
| Angiogenesis | Train | CRC | GSE146771 [13] | 2155 | Macrophage | 551 | 1604 |
| Macrophage M1 polarization | Train | KIRC | GSE111360 | 9746 | Macrophage | 5180 | 4566 |
| Macrophage M2 polarization | Train | KIRC | GSE111360 | 9746 | Macrophage | 4566 | 5180 |
| Inflammation | Train | CDC | GSE154763 [14] | 5461 | Dendritic cells | 1928 | 3533 |
| vCAF signature | Train | ICC | GSE142784 [15] | 6475 | Fibroblast | 1685 | 4790 |
| mCAF signature | Train | ICC | GSE142784 | 6475 | Fibroblast | 638 | 5837 |
| apCAF signature | Train | ICC | GSE142784 | 6475 | Fibroblast | 3051 | 3424 |
| iCAF signature | Train | ICC | GSE142784 | 6475 | Fibroblast | 379 | 6096 |
| Progenitor CAF | Train | FibroXplorer human | NA | 10,355 | Fibroblast | 1979 | 8376 |
| Progenitor CAF | Train | Mouse melanoma | Under submission | 4594 | Fibroblast | 3005 | 1589 |
| Progenitor CAF | Validation | FibroXplorer mouse | NA | 99,596 | Fibroblast | 12,453 | 87,143 |

*Notes*: KIRC, kidney clear cell carcinoma; HCC, hepatocellular carcinoma; NSCLC, non-small cell lung cancer; PDAC, pancreatic ductal adenocarcinoma; HGSOC, high-grade serous ovarian cancer; LIHC, liver hepatocellular carcinoma; UCEC, endometrioid cancer; CRC, colorectal cancer; LUNG, lung cancer; CDC, classic dendritic cells; ICC, intrahepatic cholangiocarcinoma; BRCA, breast cancer; CAFs, cancer-associated fibroblasts; vCAF, vascular cancer-associated fibroblasts; mCAF, matrix CAFs; apCAF, antigen-presenting CAF; iCAF, inflammatory CAFs.

**References**

[1] Neal JT, Li X, Zhu J, Giangarra V, Grzeskowiak CL, Ju J, et al. Organoid modeling of the tumor immune microenvironment. Cell 2018;175:1972–88.e16.

[2] Zheng C, Zheng L, Yoo JK, Guo H, Zhang Y, Guo X, et al. Landscape of infiltrating T cells in liver cancer revealed by single–cell sequencing. Cell 2017;169:1342–56.e16.

[3] Guo X, Zhang Y, Zheng L, Zheng C, Song J, Zhang Q, et al. Global characterization of T cells in non–small–cell lung cancer by single–cell sequencing. Nat Med 2018;24:978–85.

[4] Lin W, Noel P, Borazanci EH, Lee J, Amini A, Han IW, et al. Single–cell transcriptome analysis of tumor and stromal compartments of pancreatic ductal adenocarcinoma primary tumors and metastatic lesions. Genome Med 2020;12:80.

[5] Hu Z, Artibani M, Alsaadi A, Wietek N, Morotti M, Shi T, et al. The repertoire of serous ovarian cancer non–genetic heterogeneity revealed by single–cell sequencing of normal fallopian tube epithelial cells. Cancer Cell 2020;37:226–42.e7.

[6] Darmanis S, Sloan SA, Croote D, Mignardi M, Chernikova S, Samghababi P, et al. Single–cell RNA–seq analysis of infiltrating neoplastic cells at the migrating front of human glioblastoma. Cell Rep 2017;21:1399–410.

[7] Neftel C, Laffy J, Filbin MG, Hara T, Shore ME, Rahme GJ, et al. An integrative model of cellular states, plasticity, and genetics for glioblastoma. Cell 2019;178:835–49.e21.

[8] Zhang Q, He Y, Luo N, Patel SJ, Han Y, Gao R, et al. Landscape and dynamics of single immune cells in hepatocellular carcinoma. Cell 2019;179:829–45.e20.

[9] Wu TD, Madireddi S, de Almeida PE, Banchereau R, Chen YJ, Chitre AS, et al. Peripheral T cell expansion predicts tumour infiltration and clinical response. Nature 2020;579:274–8.

[10] Savas P, Virassamy B, Ye C, Salim A, Mintoff CP, Caramia F, et al. Single–cell profiling of breast cancer T cells reveals a tissue–resident memory subset associated with improved prognosis. Nat Med 2018;24:986–93.

[11] Wang H, Gong P, Chen T, Gao S, Wu Z, Wang X, et al. Colorectal cancer stem cell states uncovered by simultaneous single–cell analysis of transcriptome and telomeres. Adv Sci (Weinh) 2021;8:2004320.

[12] Habermann AC, Gutierrez AJ, Bui LT, Yahn SL, Winters N, Calvi CL, et al. Single–cell RNA sequencing reveals profibrotic roles of distinct epithelial and mesenchymal lineages in pulmonary fibrosis. Sci Adv 2020;6:eaba1972.

[13] Zhang L, Li Z, Skrzypczynska KM, Fang Q, Zhang W, O’Brien SA, et al. Single–cell analyses inform mechanisms of myeloid–targeted therapies in colon cancer. Cell 2020;181:442–59.e29.

[14] Cheng S, Li Z, Gao R, Xing B, Gao Y, Yang Y, et al. A pan–cancer single–cell transcriptional atlas of tumor infiltrating myeloid cells. Cell 2021;184:792–809.e23.

[15] Zhang M, Yang H, Wan L, Wang Z, Wang H, Ge C, et al. Single–cell transcriptomic architecture and intercellular crosstalk of human intrahepatic cholangiocarcinoma. J Hepatol 2020;73:1118–30.
