# Supplementary figures and images for: SuperFeat: Quantitative Feature Learning from Single-cell RNA-seq Data Facilitates Drug Repurposing
Source: Genomics Proteomics Bioinformatics. 2024 May 23;22(3):qzae036. doi: 10.1093/gpbjnl/qzae036 (PMC12016572; doi:10.1093/gpbjnl/qzae036)

Training data: KIRC

(Exhaustion: CD8Tex)


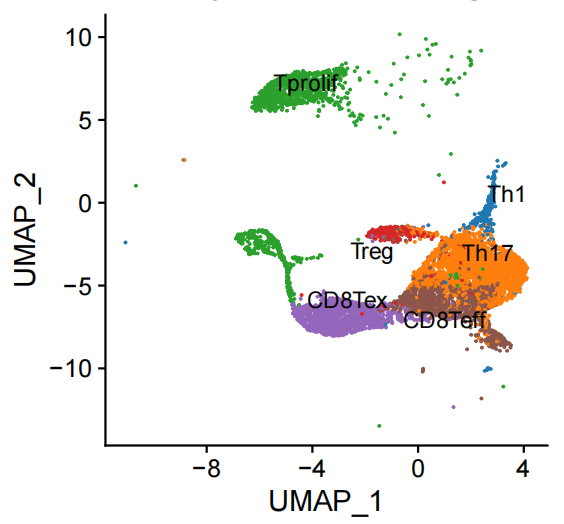

Supplement: qzae036_Supplementary_Data [file qzae036_supplementary_data.zip › Figure S1-done.docx]

Validation data: HCC

(Exhaustion: C4_CD8-*LAYN*)


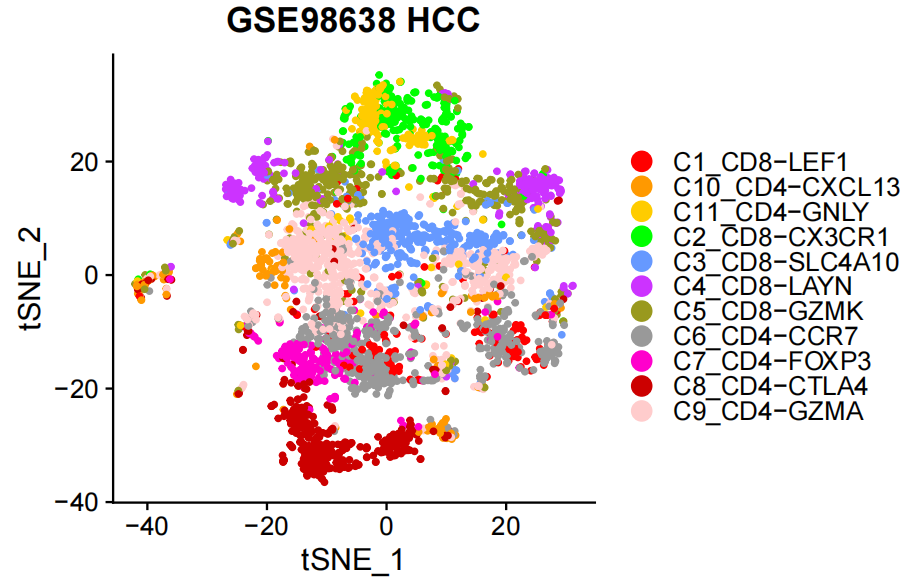


_


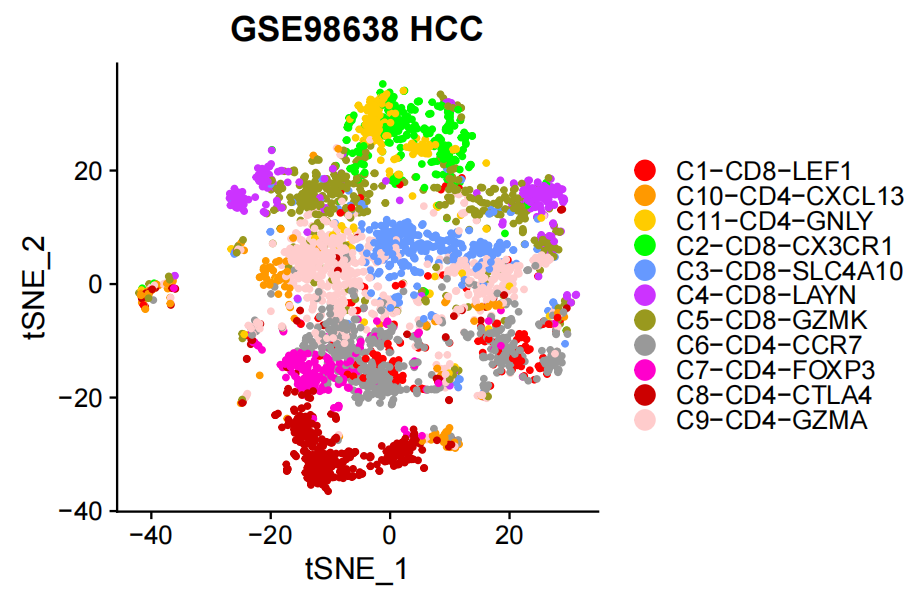

Supplement: qzae036_Supplementary_Data [file qzae036_supplementary_data.zip › Figure S2-done.docx]

**A**


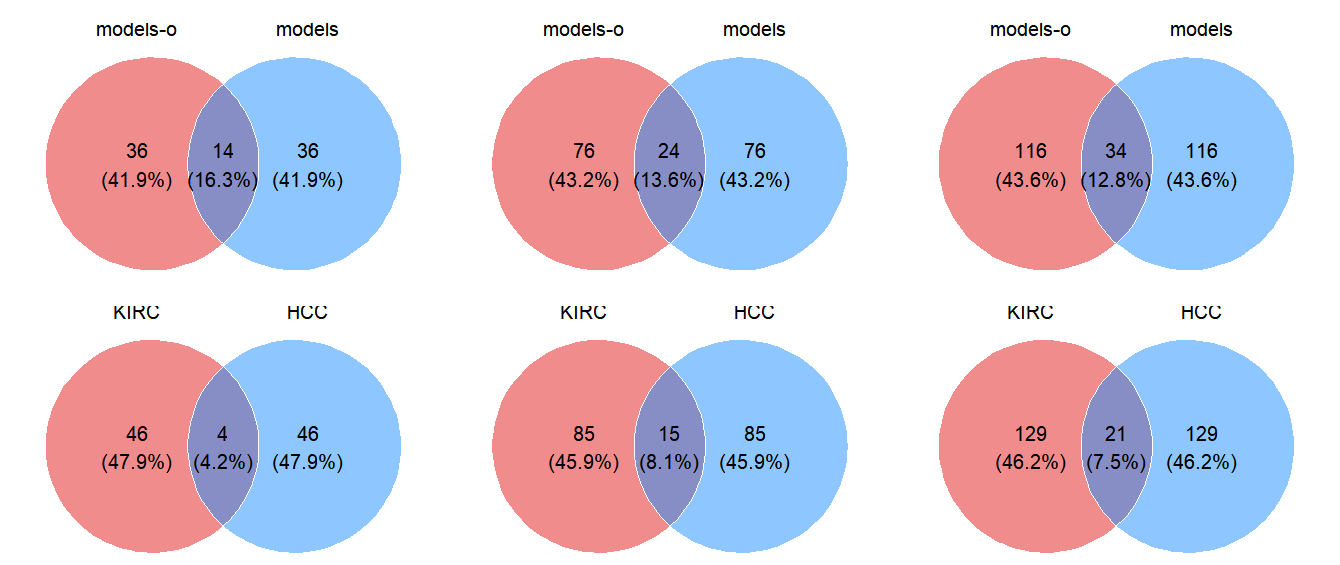

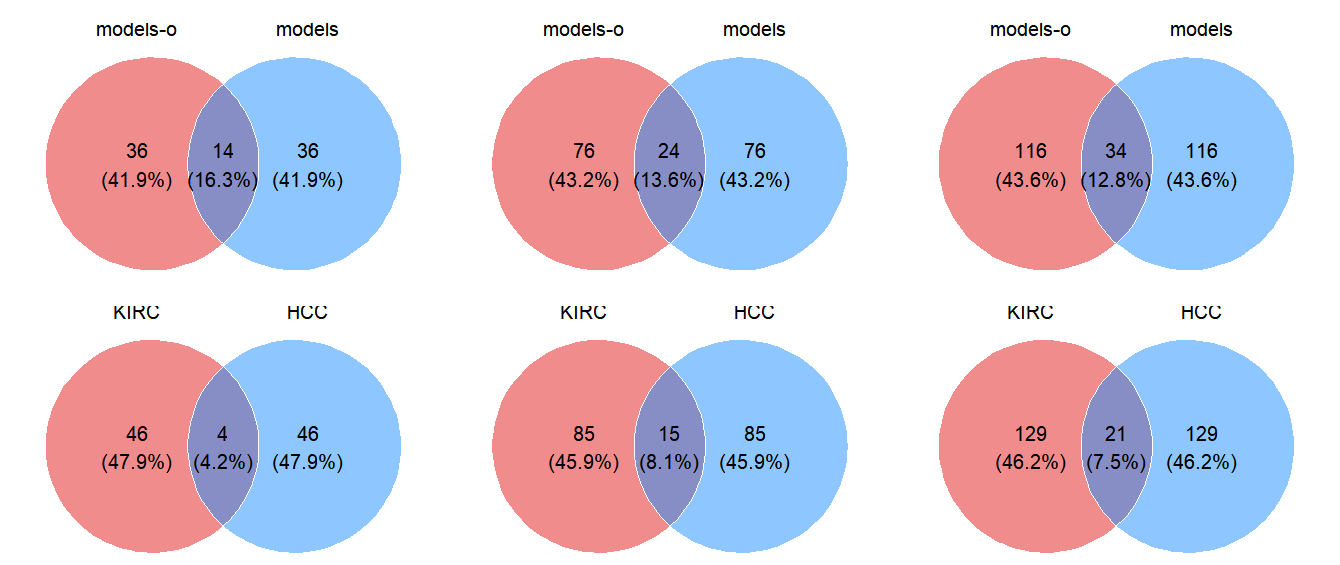


**B**

**Top 150**

**Top 100**

**Top 50**

GSE111360

GSE98638

**GSE98638**

**GSE111360**


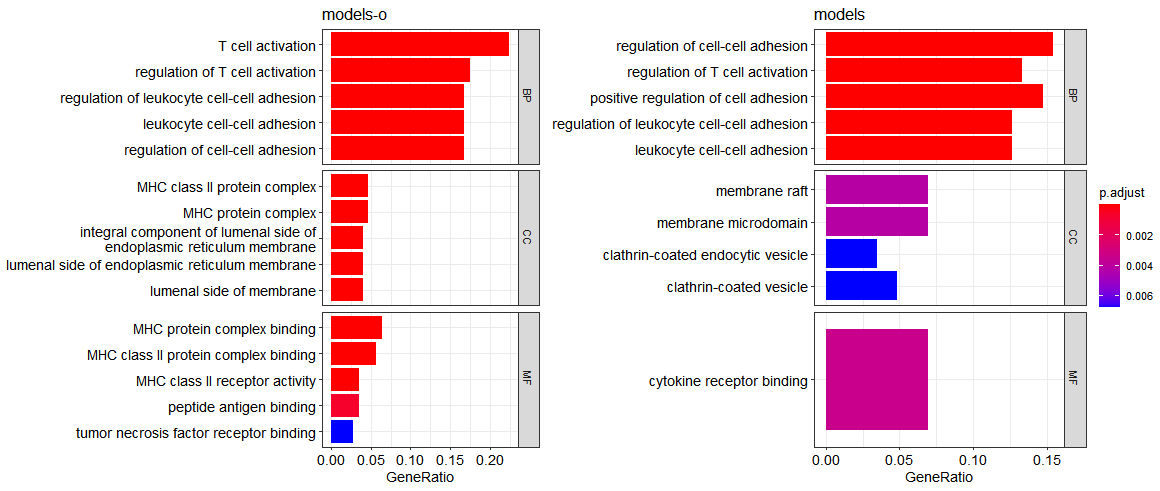

Supplement: qzae036_Supplementary_Data [file qzae036_supplementary_data.zip › Figure S5-done.docx]

**A**

**B**

**Top 150**

**Top 100**

**Top 50**


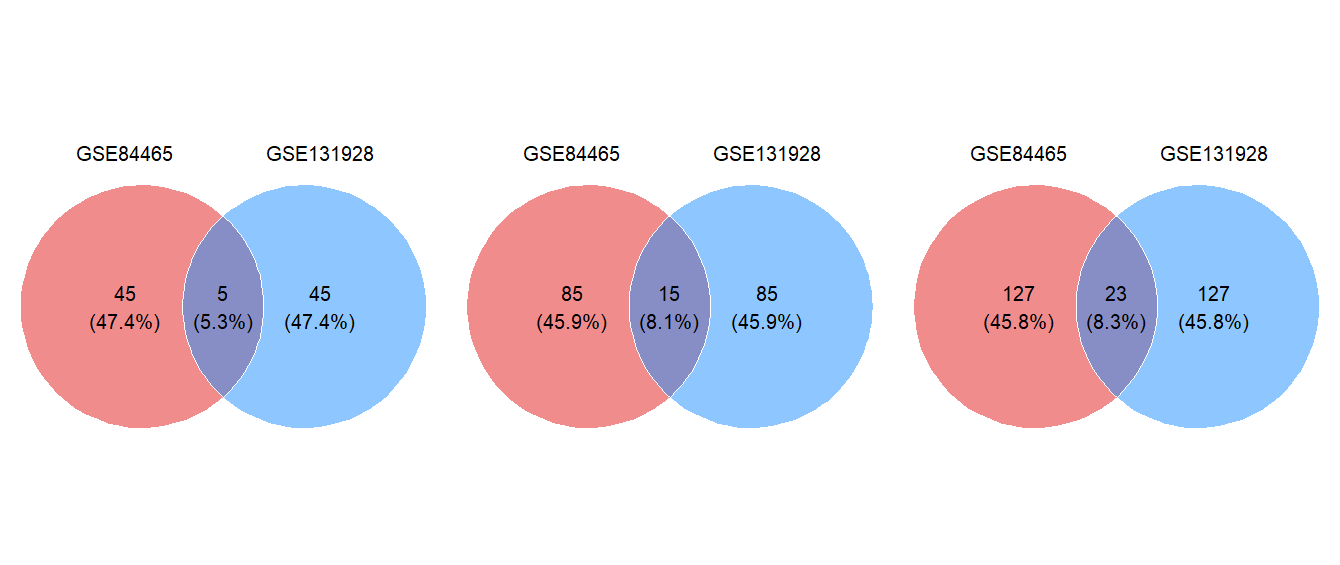

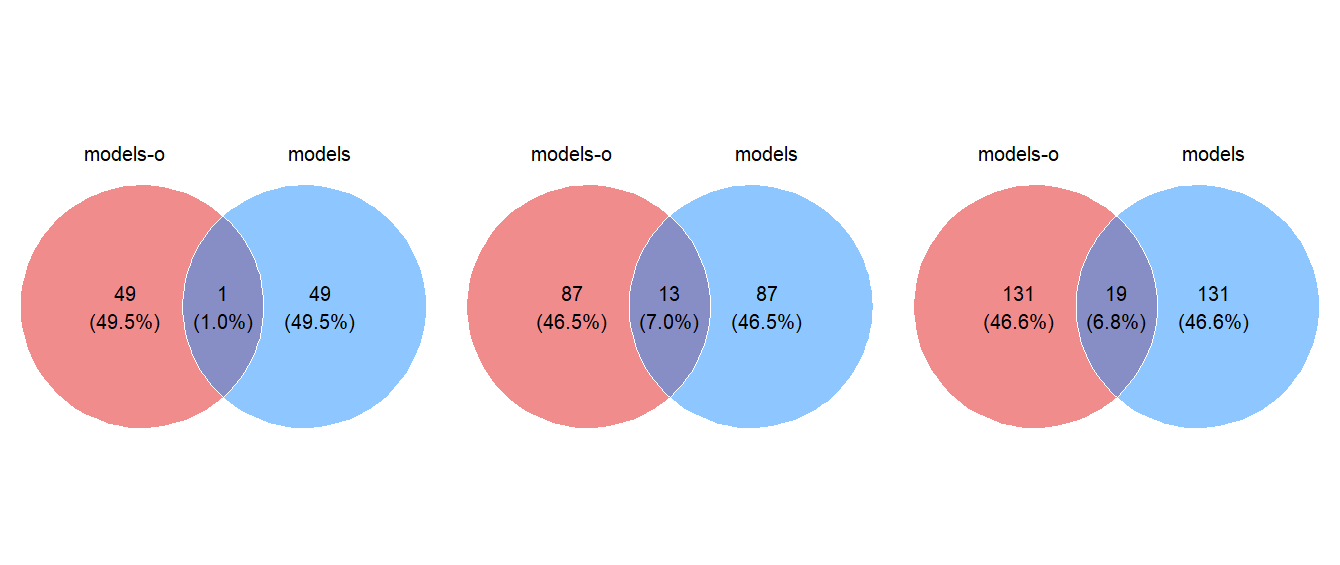


GSE84465

GSE131928

**GSE131928**

**GSE84465**


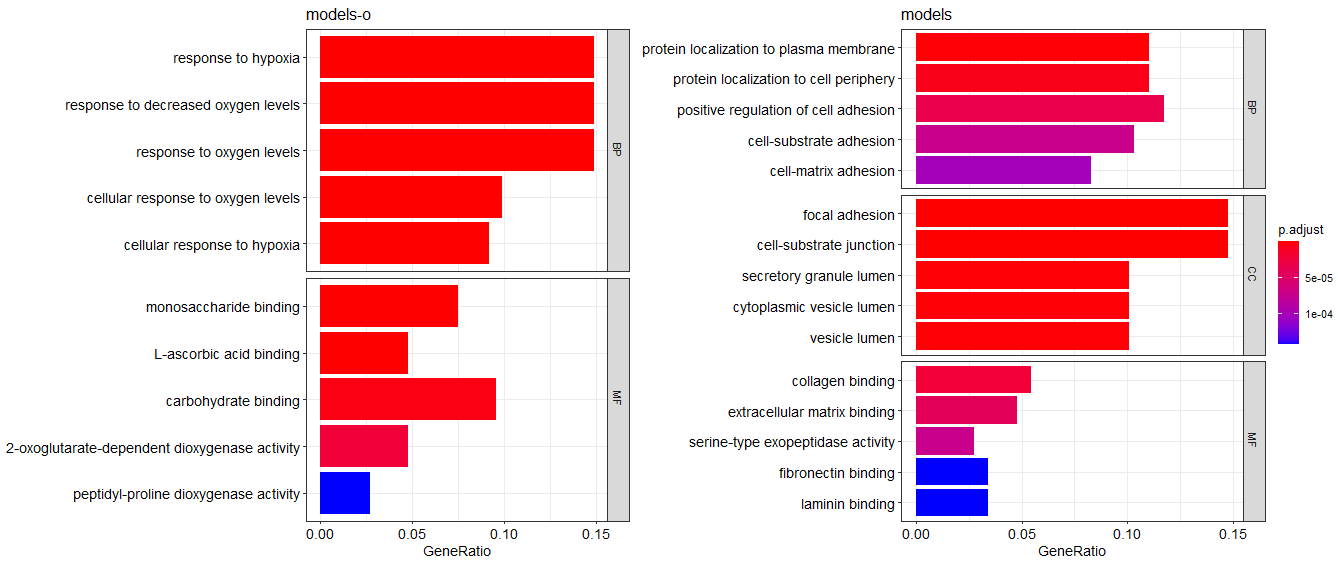

Supplement: qzae036_Supplementary_Data [file qzae036_supplementary_data.zip › Figure S6-done.docx]
